# Use of the perceptual point-spread function to assess dysphotopsias

**DOI:** 10.1371/journal.pone.0306331

**Published:** 2024-07-19

**Authors:** Krzysztof Petelczyc, Jan Bolek, Karol Kakarenko, Karolina Krix-Jachym, Andrzej Kołodziejczyk, Marek Rękas

**Affiliations:** 1 Faculty of Physics, Warsaw University of Technology, Warsaw, Poland; 2 Ophthalmology Department, Military Institute of Medicine, Warsaw, Poland; Technical University of Munich, Germany, UNITED KINGDOM OF GREAT BRITAIN AND NORTHERN IRELAND

## Abstract

Nowadays many patients are choosing EDOF or multifocal lenses for replacement of natural lens in cataract surgery. This can result in issues such as presence of dysphotopsias, namely halo and glare. In this work, we propose a new perimetry method to describe dysphotopsias in far-field region in a presence of bright, point-like light source. We constructed a custom device and designed measurement procedure for quantitative measurement of dysphotopias in the center of visual field and used it to examine patients with mild cataracts or implanted IOLs. Our approach may help in establishing an objective method to study and compare dysphotopsias.

## Introduction

Sun glare is one of the main risks associated with driving a car during the summer [1]. Similar conditions, such as glare or halos, are experienced by people affected by positive dysphotopsia during night-time traffic.

Positive dysphotopsia is described by patients as the appearance in the visual field of light streaks, light arcs, central flashes, and starbursts that are induced by an external light source [2]. As opposed to the bright manifestations in positive dysphotopsia, negative dysphotopsia is often described by patients as a dark shadow or crescent, usually in the temporal visual field [3]. Therefore, dysphotopsia is a condition in which the eye visualizes illusory light at a point of the visual field where there is no light source (positive) or visualizes no (or visualizes poorly) light at a point of the visual field wherein there is a light source (negative). Certain aspects of dysphotopsia have been observed in 49% of patients at some time after refractive surgery [4].

These effects may be attributed to physiological, neurological, or optical causes. The design of the edge of the intraocular lens, the refractive index of the optical material, and the design of the optics have been mainly implicated as the major contributing factors to positive dysphototopsia, especially when there is an oblique light source in the field of view [2]. However, even in direct light conditions, illusory light distributions may result from corneal surface irregularities, astigmatism, lens tilt, lens decentration, or opacification of the ocular media (e.g., cataract) [5]. Positive dysphototopsia may also be a consequence of the deliberate optical properties of multifocal lenses to create two or more light foci along the optical axis [6]. This lens type enables the simultaneous, high-resolution imaging of objects from several designed distances on the surface of the retina. A focused image of a point-like object placed at one of those distances is then blurred by the defocused overlapped images from other distances.

In this study, we focus on positive dysphotopsias caused by direct light in the center of the field of view. We also limit our considerations to the simplest illusory light distributions with rotational symmetry. In the presence of a point-like light source, an intensely bright spot (glare) appears at the center surrounded by rings of light (halo) [6]. In daylight conditions, the symptoms may be less noticeable or bothersome compared with night-time or low-light situations, but they can still affect one’s overall visual comfort and quality of life. However, a night vision problem arises when scenes consist of point-like light sources (street lights, car lights, and reflections) with high-background contrast. Visible glare and halo phenomena noticeably increase the threshold brightness of objects near bright points within the field of view. Therefore, these phenomena clearly limit the safety of road traffic participation and the comfort of vision in the dark [7].

Owing to their good distant vision and low incidence of photic phenomena, including halo and glare, monofocal intraocular lenses (IOLs) remain the most commonly implanted lenses [8]. By contrast, more than one-third of patients receiving multifocal IOLs experience photic phenomena [9]. To overcome these problems, there is an increasing interest in emerging technologies that can enhance the performance of monofocal IOLs and reduce the undesirable photic phenomena of multifocal IOLs. Extended depth-of-focus (EDOF) IOLs assume a continuous focal range over most distances [10]. These IOLs generate various overlapping focal points to produce the effect of a continuous extended focus [11, 12]; however, they do not fully resolve the halo and glare problems [13].

Some solutions are dedicated to the problem of measuring and assessing the halo and glare phenomena. The first approach is based on the optical parameterization of the point-spread function (PSF) and modulation-transfer function (MTF) of the lenses and the visualization of undesirable aberrations [14]. This approach requires the use of an eye model, which is always the result of certain simplifications and idealizations, such as the sphericity and centration of refractive surfaces. The optical parameters of the eye can be also measured in vivo using the double-pass, optical integration method. [15] Designed instruments perform stray-light measurements in an angular range of 3–8° to estimate the single stray-light parameter of the measured eye [16]. These approaches do not consider the physiological processes related to vision, which may (collectively) lead to problems with night vision. Nevertheless, they allow the objective prediction of the occurrence of glare and halo phenomena and associate them with the specific design of a corrective lens and possible (modeled or measured) eye aberrations. It also enables the measurement of the size and intensity of photic phenomena using various defined metrics.

The second approach assessed the subjective perception of halo and glare phenomena among patients with implanted multifocal lenses (Halo & Glare Simulator; Eyeland-Design Network GmbH, Vreden, Germany) [17]. These analyses are often based on questionnaires in which respondents define the type of visual disturbance (from a closed graphic list) and its level (on a quantitative scale). Assessing the visual sensation on an interval scale is difficult and imprecise. Therefore, it is necessary to develop a more reliable quantification method for the size and intensity of undesirable phenomena in the field of view [18] that should be based on a psychophysical methodology.

One of the methods that combines the objectivity of the study with the subjectivity of the patient’s impressions is based on the commercially available Oculus C-quant device (Oculus Optikgeräte, GmbH, Wetzlar, Germany) used for the diagnostic measurement of stray light in night vision [19]. The compensatory comparison method implemented in the device involves the identification of an appropriate intensity of the flickering area located at the center of the field of view to compensate for the glare caused by a bright ring flickering in the counter phase. Appropriate selection of light intensities at the center of the visual field makes the glow from the ring visible for half a period, and targets with the same brightness become visible for the second half of the period; thus, flickering disappears. The disadvantage of this method is that the result is in the form of a single marker, which makes it difficult to parameterize the halo and glare phenomena precisely and independently.

Another method used in a MonCv3 device (Metrovision, Pérenchies, France) is an approach in which the patient reads optotypes (with a specific contrast) placed at an increasing distance from a point light source [20]. Because the light source creates glare and a halo in the field of view of the examined person, the minimum brightness of the identifiable individual optotypes differs depending on the distance from the source. The measurement of this brightness enables parameterization with a relatively low-spatial resolution of the glare and halo phenomena in the form of their profiles and intensities. C-quant and MonCV3 devices are used clinically to diagnose cataracts, glares, and halo levels [21].

In contrast to the precise method of near-field perimetry offered by commercial perimeters, which is part of almost every ophthalmologic practice, the available methods of stray-light parameterization provide limited information regarding the perceived light distribution [22].

In this study, we present and verify a simple central far-field perimetry method based on psychophysical adaptation of the optical point spread function (PSF) concept. We quantified light perception at a discrete set of points (characterized by polar coordinates) in the visual field. To induce photopic phenomena, we used a point-like, strong light source in the center of the visual field on which patients fixated. Contrary to other qualitative research methods, which defined only the type of photopic phenomenon and its simple characteristics, the proposed solution determines precisely and objectively the parameters and intensity of the perceived stray light that causes some dysphotopsias. To obtain an optimal precision-to-examination time ratio, we limited the resolution and assumed rotational symmetry of the photopic phenomena, i.e., we considered only the perceived halo and glare effects. These conditions reliably emulate real night vision and allow physicians to measure and reconstruct accurately what the patient sees.

## Material and methods

Our approach was based on measuring the properties of optical imaging systems. Because in Fourier optics the PSF describes the quality of an image formed by a lens, we conducted a similar experiment using the human eye’s lens as the test lens and human visual perception as the signal receiver.

### Patients

Participants were recruited from 1st April till 30th June 2022 among the patients at the Military Institute of Medicine in Warsaw and among patients of ophthalmologists working in private practice. Written informed consent was obtained from all the participants. This study adhered to the principles of the Declaration of Helsinki and was approved by the Bioethical Committee of the Military Institute of Medicine in Warsaw, Poland. The study protocol was registered at ClinicalTrials.gov (NCT05516160).

The exclusion criteria of the eyes qualified for the test were such as Fuchs dystrophy and other corneal diseases, evidence of severe eye disease, ophthalmic surgeries other than cataract surgery, clinically active or past uveitis, intraocular pressure (IOP) >21 mmHg, glaucoma, retinal detachment or its suspicion in ultrasound examination of the eyeball, systemic diseases with ocular symptoms including diseases that may affect the function of corneal endothelial cells. We did not consider patients who had intolerance of the examination in the slit lamp or other procedures planned in the examination, pregnancy, mental disorders or emotional instability to an extent that does not allow the subject’s informed consent in the study and presence at scheduled follow-up visits, documented sensitivity to pharmacological agents used in the study, i.e. topical anesthetics, fluorescein, other related to the ophthalmological examination fatal severe illnesses or a patient’s medical condition preventing the study from continuing for a period of 6 months, current participation in other research programs and therapy with oral anticoagulants.

Measurements were conducted in both eyes of 12 healthy adult patients (seven male, five female) ranging in age from 39 to 78 years. The patients were divided into subgroups: those with a crystalline lens (two eyes), cataracts (seven eyes), or implanted IOLs (monofocal in eight eyes and multifocal/EDOF in seven eyes). Refractive errors were corrected before the examinations of all the eyes. The best corrected distance visual acuity (BCDVA) of 1.0 was observed in all patients, except for the subjects who suffered from cataracts. The patients did not have a history of ophthalmic surgery, except for cataract removal in the examined eyes, and had no pre-existing eye diseases that could impact the test results.

Dysphotopsia analyses were performed on the five commercially available IOLs: Hoya iSert 254-e (HOYA Surgical Optics, Chromos, Singapore): six eyes; Acrysof SA60AT: three eyes, Acrysof IQ Vivity (both Alcon, Geneva, Switzerland): one eye, RayOne EMV (Rayner, West Sussex, United Kingdom): four eyes, and Tecnis Symfony ZXR00 (Johnson & Johnson Vision, Irvine, CA, USA): two eyes. In addition, seven eyes before cataract surgery and two healthy (reference) eyes were also investigated.

Hoya iSert 254-e and Acrysof SA60AT are monofocal, aspherical, hydrophobic, acrylic, single-piece IOLs [23, 24]. The RayOne EMV is a nondiffractive, hydrophilic, acrylic, aspheric, single-piece IOL that induces a controlled positive spherical aberration spreading the light along the visual axis, elongating the focal range from a far to an intermediate range with a depth of focus of up to 1.5 D (per lens on the spectacle plane) [25]. The Acrysof IQ Vivity is a nondiffractive, hydrophobic, acrylic single-piece EDOF IOL, that has a negative spherical aberration on the anterior surface that compensates for the positive spherical aberration of the cornea [26]. Instead of splitting light into multiple vision zones as in traditional multifocal IOLs, Vivity uses a central optical element to change the shape of the wavefront. Tecnis Symfony ZXR00 is a hydrophobic, acrylic, single-piece, combined diffractive-refractive-depth-of-focus IOL with an aspheric anterior surface that results in negative spherical aberration [27]. Instead of focusing, ZXR00 expands the depth of focus across the principles of the Echolette diffractive ring, designed to enhance the intermediate VA.

We present also a detailed case study of a 66-year-old female patient (LYS) who underwent treatment in the right eye and developed untreated cataract in the left eye. These conditions allowed for comparison of the vision in the eyes before and after the surgical procedure using our method. LYS visited the hospital approximately 1 year before the analysis because of the deterioration of her vision in both eyes. The patient had no history of ophthalmological treatments. The BCDVA scores obtained before surgery were 0.8 logMAR in the right eye (OD) and 0.3 logMAR in the left eye (OS). The intraocular pressure was 19 mmHg and 18 mmHg. Slit-lamp examination revealed a mature senile cataract in the OD and an immature senile cataract in the OS. Preoperative corneal astigmatism values of -0.50 Dcyl and -1.0 Dcyl were obtained for the OD and OS, respectively. A fundus examination revealed no insights into the OD and a normal ocular fundus in the OS. Ultrasonography revealed that the retinas had adhered to both eyes. The axial length of the eyeball was 23.03 mm in the OD and 23.07 mm in the OS. The LYS qualified for cataract removal in the OD with the implantation of an IQ Vivity intraocular lens (IOL). After the phacoemulsification of the cataract with IOL implantation, BCVA improved in the operated eye; the achieved BCVA was 0.0 logMAR and the intraocular pressure remained normal. No postoperative abnormalities were observed in the ophthalmological examination.

### Design of the measurement device

**Fig 1:**
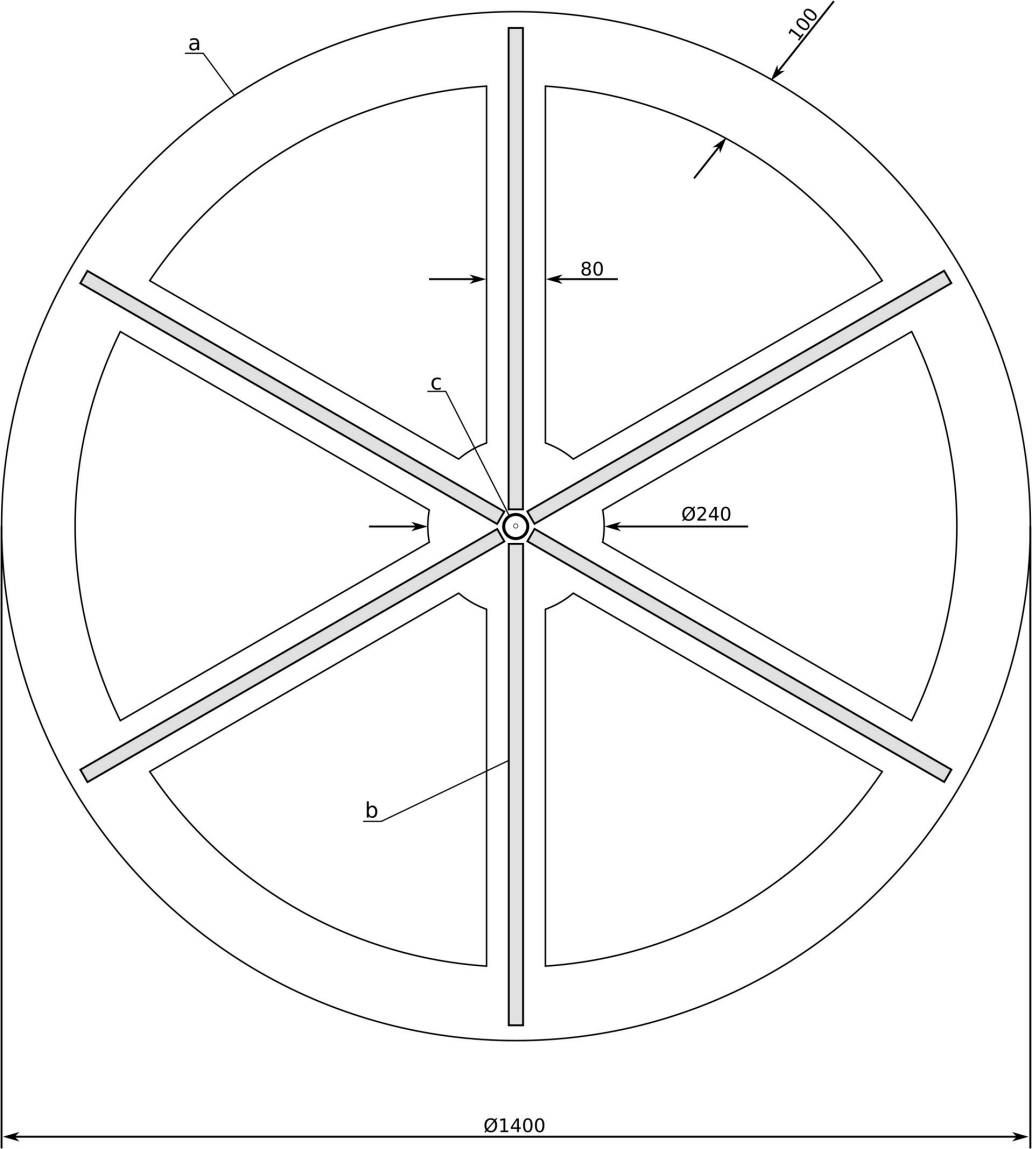
Constructed device displaying the stimuli in experiments (patent pending). Patients were located 5 m in front of the device. a) frame, b) (gray stripes) ‐ probe diodes (0–1.3 cd) in different directions and distances, and c) central white diode (40 cd) causing dysphotopsia. Marked dimensions are given in millimeters.

**Fig 2:**
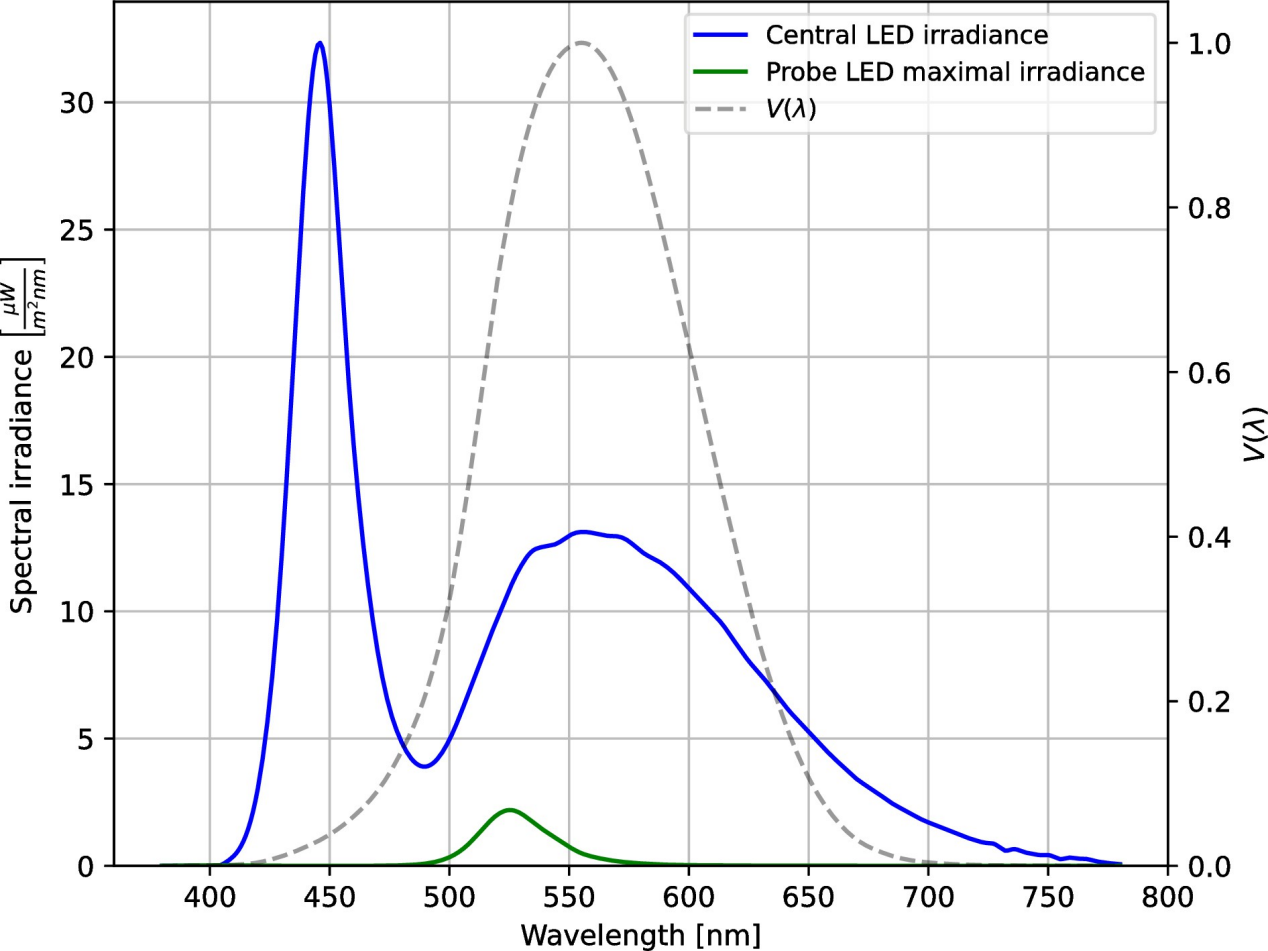
Spectral irradiance of used light sources and photopic luminous efficiency function V(λ). Blue curve corresponds to spectral irradiance of the central white phosphor-based diode which mimics light spots in the night vision (eg. modern car lights, diode-based street lamps). Green curve represents the spectral irradiance of green probe diodes at maximum light intensity (their intensity was controlled by an algorithm to find just noticeable luminosity for each patient). Dashed line represents photopic luminous efficiency function V(λ) established by the Commission Internationale de l’Éclairage (CIE).

In order to perform these measurements, a dedicated experimental device (patent no. PL440849) was built (Fig 1). This device contained a central phosphor-based white diode (40 cd) and six arms equipped with probe diodes that acted as threshold detectors at different points in the visual field. We used green light-emitting diodes (LEDs) (λ = 522–525 nm) with a controlled luminous intensity of up to 1.3 cd (± 1,5%) as probe stimuli. Spectral irradiance distributions of these light sources and photopic luminous efficiency function V(λ) are presented in Fig 2. The constructed device was equipped with triple red–green–blue (RGB) diodes, among which the green one had the best linear characteristics and the widest luminous intensity range. Moreover, the human eye also has the best sensitivity to green light [28].

We assumed far-vision conditions, placed the patient at a distance of 5 m, and used a 15° central field of view; diodes were placed in such a way to achieve a density of six LEDs per degree. The patients had a remote control at their disposal with six buttons related to the directions of the six arms of the device. In this manner, they could indicate the arm in which the proper stimulus was presented. Two numbers were assigned to each diode: the arm index (1–6, starting at the top center arm and increasing clockwise) and the position on each arm (1–92, starting from the nearest diode to the center diode).

### Calibration of brightness levels

First, we created a set of stimulus brightness levels that were evenly distributed based on nonlinear human perception of brightness, as opposed to the linear scale of the diode’s driving signal. To achieve objective control over perception levels, we utilized a closed set of six responses based on the M-alternative forced choices (M-AFC) method, which was presented to the patient [29]. Consequently, the uncertainty of the correct answer was determined to be 17% (the reciprocal of M).

**Fig 3:**
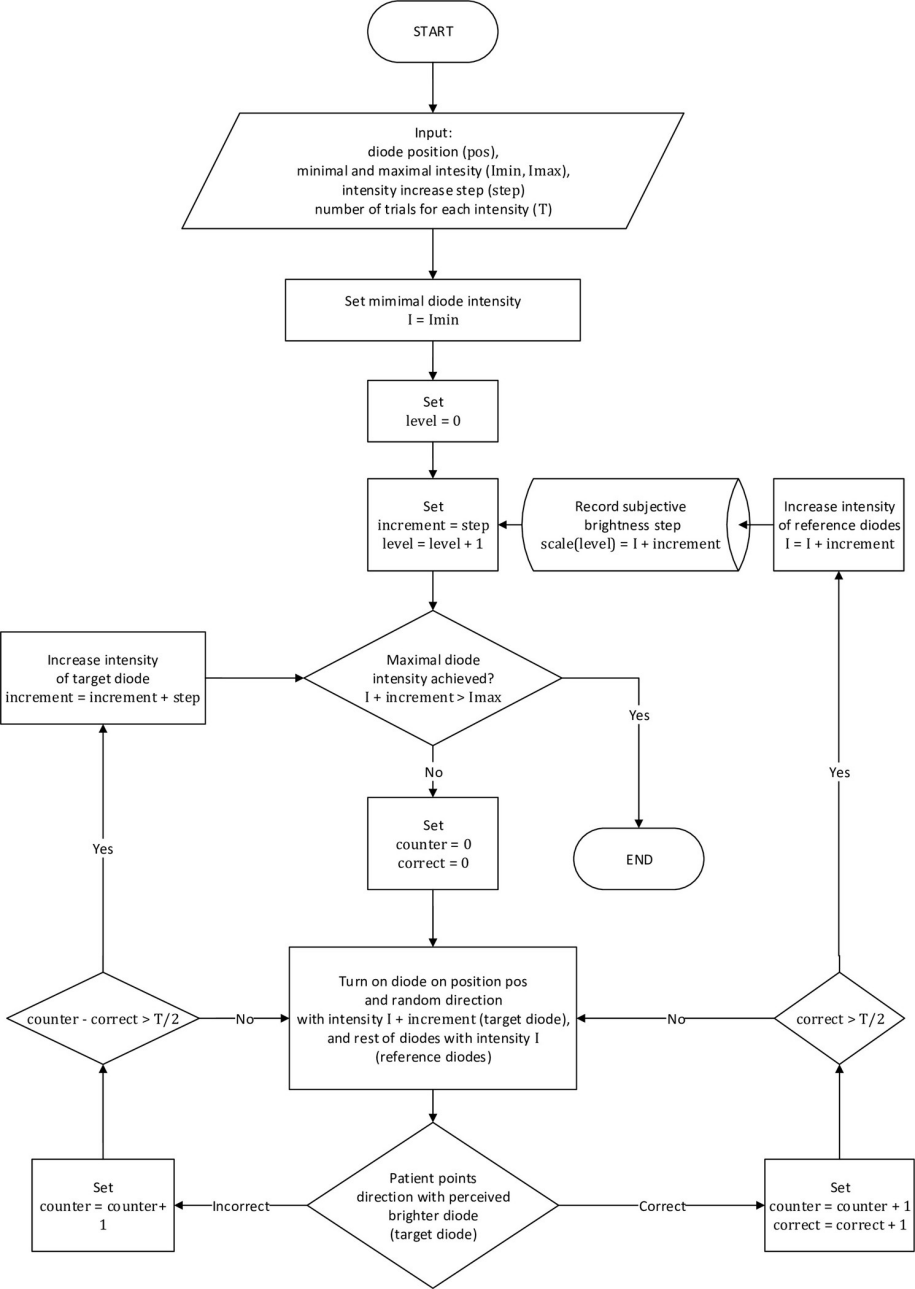
Algorithm of the brightness scale calibration experiment. In the algorithm, the following variables were used: “I” denotes the current luminous intensity of the reference diode, “inc” denotes the current difference between reference and target diode luminous intensity; “cnt” is the current number of performed trials, while “correct” is a counter of correct answers in cnt trials, both of them are counted separately for every settings of I and inc. The input parameters were set to: the angular distance of reference and target diodes from the central diode: pos = 1°; minimal and maximal luminous intensity of diodes: Imin = 0 cd, Imax = 1.3 cd, unit of inc increment: step = 5 mcd, and maximal number of trials before the change of target diode luminous intensity: T = 5. The “level” denotes the number of perceptually equal units of brightness, while the scale is an array storing brightness vs. luminous intensity function values.

We conducted the calibration experiment based on the classic “just noticeable differences” approach proposed by Weber [30]. Six probe diodes were used on the constructed device and placed in the center of the tested visual field at a distance of 5 m. Each diode was placed on a different arm at a position corresponding to 1.5° from the center of the visual field. Five diodes had the same intensities (differing by no more than 1.5%), referred to as the “reference diodes,” while in each step, one diode with a higher intensity was randomly selected and served as the “target diode.” The intensity of the “target diode” was increased until the patient correctly indicated that the diode was brighter, that is, consciously perceived the brightness difference. This corresponded to the new threshold for correct perception. The patient responded to a small keyboard controller by selecting one among six buttons, corresponding to each possible arm direction. To minimize the impact of guessing, the patient needed to identify correctly the LED’s position at least three times (out of five trials). The test was performed using the maximum possible stimulus intensity provided by the device. The calibration algorithm is presented in detail in Fig 3.

A calibration function, that is, a diode driving-signal-scale conversion to an interval perception scale, was obtained by processing the scores recorded from healthy eyes at the BCDVA condition (age 25.0 ± 5.2 years; VA: 0.0 logMAR or better, contrast sensitivity (CS): 2.0 on the Pelli–Robson scale). In all results, the diode control signal was converted to luminous intensity by the documentation of the technical properties of the diodes. The experiment was performed 10 min after adaptation to the dark without any additional light sources (background illumination less than 10 mlux).

To determine the effects of calibration, we defined 16 levels of diode luminous intensities equally distributed in the range of the subjects’ brightness perception. A detailed description of the calculations is provided in the Results and Discussion sections. The limited number of levels was related to the need to design a test protocol that lasts an optimal time for dysphotopsia measurements and ensures a reliable relation between the brightness scale and possible diode-driving signals.

### Dysphotopsia measurements

The next step involved the design of a protocol for the main experiment, including the patient’s task, a stimulus presentation strategy, and methods for the analysis of the collected data.

All experiments were performed during the same period of the day, between 10 AM and noon. First, patients were adapted to darkness over a 10 min period (background illumination less than 10 mlux). Then, at the central part of the device shown in Fig 1, a strong diode was lit to induce the perception of dysphotopsia in the subject. Then, patients had an additional one minute to adapt. After that probe diodes were used to measure the noticeable threshold brightness levels according to a scale defined during calibration. Taking into account the optimal time of patient examination (chosen to be less than 15 min) we chose 10 distances from the central diode and obtained a spatial resolution measurement of 50 arcmin. Technically, we could use all 92 diodes distributed from 0.24° to 7.67° from the central point. However, this precision increase required at least one additional minute for each diode to complete the experiments. The luminous intensities of diodes were controlled according to the adaptive Ψ (PSI) method [31] to minimize the duration of the experiment (avoiding patient frustration and fatigue) and maximize efficiency. Using this method, we assessed the threshold value to increase accuracy. For a given angular position, we conducted 20 trials to improve the reliability of the results. In each trial, we used the same M-AFC answer-acquisition method as described previously.

**Fig 4:**
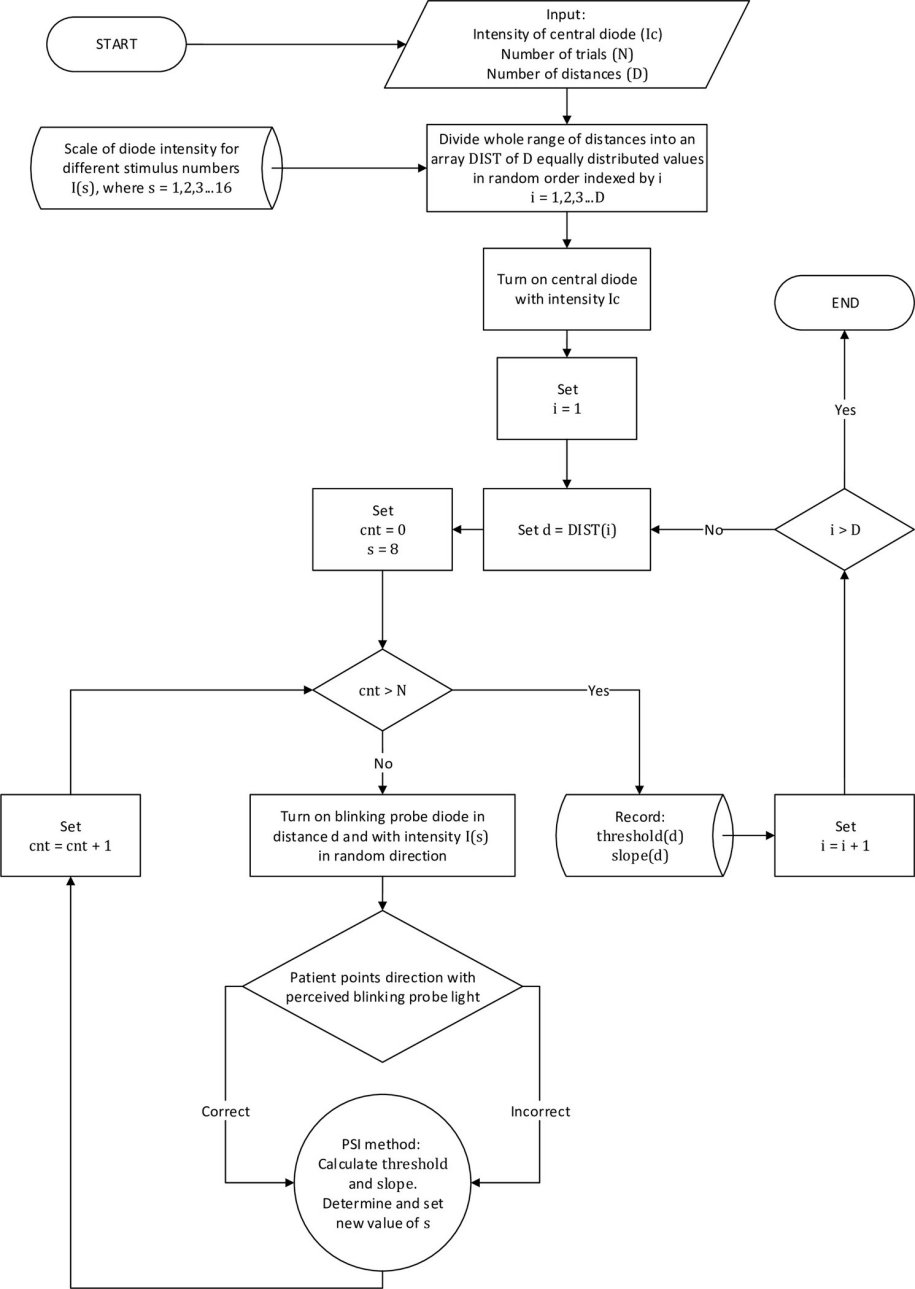
Algorithm of the main experiment targeted to measure the perceptual point spread function (pPSF) treating patients visual system including its optics, physiology and psychology as an integrated imaging system, and patient’s perceptions as its output signal. In the algorithm, the following variables were used: “Ic” denotes the intensity of the central diode (Ic = 40 cd); “DIST(i)” is a randomly sorted list of “D” angular stimuli positions distributed equally as a function of distance from 0.24° to 7.67° from the central point (D = 10), while “i” is an index corresponding to the current distance of a probe diode (“d”); “N” denotes the number of trials for each stimuli position (N = 20); “s” denotes the perceptual brightness value transformed to diode luminous intensity by an array “I(s)” corresponds to the table “scale (level)” determined by the algorithm presented in Fig 3; “cnt” is a counter of trials for the current probe diode’s distance, array threshold (d), and slope (d), i.e., it denotes the intensity of the single point of the pPSF and its uncertainty. In each trial, a probe diode was lit at a distance d in a random angular direction (on a random arm of the device) with a brightness s determined by the PSI algorithm based on previous answers. The assigned patient task was to choose the correct direction (arm).

Each trial was a blinking green light (with a 1 s period) of the probe diode on a random arm of the device at one of 10 assumed angular distances (in random order) from the central light spot. Patients were asked to point to the direction where they perceived the blinking light originated using the same keyboard, as described previously. There were no response time limitations. In the absence of stimulus perception, patients were asked to select a random direction. The software that controlled the experiment analyzed the answers in real time and calculated the intensity of the presented stimuli and their positions based on the accuracy of the responses and the PSI algorithm. Each test consisted of 20 trials. Fig 4 shows the detailed measurement algorithm.

After a set of 10 trials at different angular positions, we constructed a map of the thresholds and created a perceptual PSF (pPSF). Each point of this characteristic was a minimum (threshold) value of the perceived luminous intensity distribution (as a function of the distance from the central point) and consisted of a bright light spot. The remaining points were fitted using spline interpolation. This approach was inspired by the optical PSF, which is commonly defined using the light-intensity distribution of the image of a point-like source. Patient’s visual system including its optics, physiology and psychology was treated as a integrated imaging system, and patient’s perceptions as its output signal. In our experiment (similar to the optical PSF determination), we assumed the rotational symmetry of a pPSF, even though it was possible to create a two-dimensional map (without postulating this assumption) by modifying the measurement procedure.

Subject to the given assumptions about resolution in the spatial and luminous intensity domains and uncertainty level, the mean time of a single set of trials was 10 min. In longer experiments, precision could be improved, as well as asymmetric photopic phenomena could be taken into account.

## Results and discussion

### Brightness levels calibration

**Fig 5:**
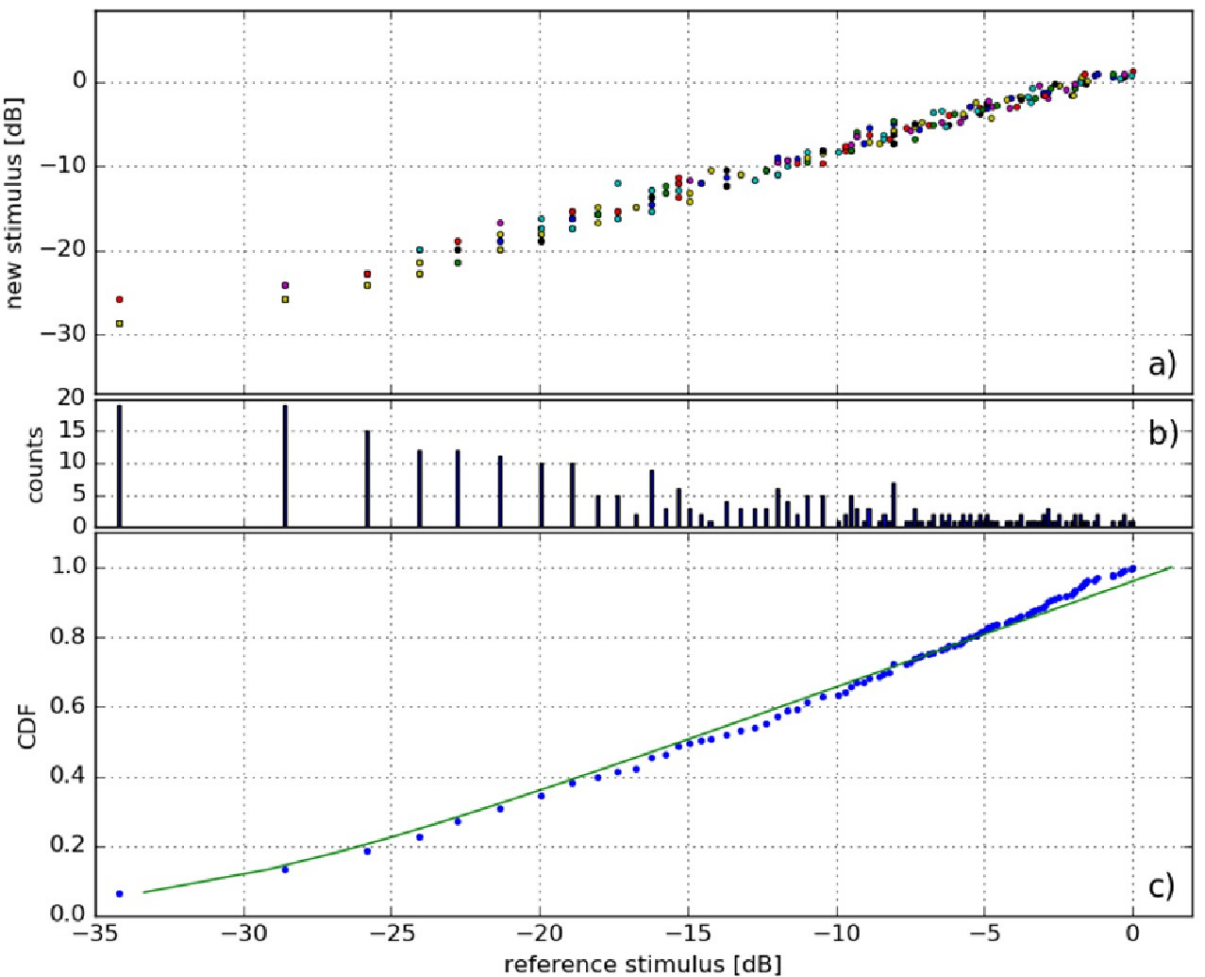
Calibration of perceptually equal brightness steps: a) plot of correct indication trial outcomes in the form of brighter diode vs. reference levels; b) plot of the counts of each level of reference diode level in a) (some points overlap); c) plot of the cumulative distribution function (CDF) of b) using a fitted calibration curve. Units (dB) correspond to $10log\left(\frac{I}{I_{max}}\right)$, where I denotes the luminous intensity and Imax denotes the maximum luminous intensity emitted by the diode probe. All values are assigned to candela units.

The calibration procedure included experimental data collection, histogram construction, and calibration function fitting. In Fig 5A the luminous intensities for all subjects’ brightness threshold values were plotted against the reference luminous intensities. During the calibration procedure, in the new step, the reference luminous intensity was equal to the luminous intensity threshold from the previous brightness level.

To transform the data from all patients into a single calibration curve, a cumulative distribution function (CDF) was determined from the histogram of the reference light intensities of the measured perceptual brightness steps (Fig 5B). The CDF was constructed by summarizing the counts and resultant sums were normalized. This enabled the averaging of the subjects’ answers (Fig 5C), accounting for the different distributions of the new threshold levels, and ensuring that the stimulus levels were distributed linearly according to human perception.

The resulting dependency of the CDF on the reference stimulus level was approximately exponential [29]. The data were fitted using the least-squares method and the resultant curve (presented in Fig 5C in green) is defined by an equation with three parameters A, B and C,

f(x)=A(exp(Bx)+C) (1)

where x is the normalized reference stimulus level equal to $10\;log\left(\frac{I}{I_{max}}\right)$. The coefficients were determined as *A* = 7.0279×10^−4^ and *B* = 7.5611. The added shift *C* = -1 took into account the fact that the sensation for stimulus *I* = 0 should also be zero.

The set of discrete stimulus levels was selected based on the fitted function. The CDF values were evenly divided into 16 levels (which correspond to 16 perceptually equally spaced stimuli). The difference between the arguments of the fitting function for any two adjacent levels became a single brightness step. Because of the luminous intensity quantization of diodes caused by the finite control signal step, the selection of these stimuli resulted in several minor errors between the values of the expected (fitted) and actual (available) values, as presented in Table 1. Dividing the brightness scale into more steps will be problematic at low-brightness levels where the successive steps of the control signal changes are wide; thus, some luminous intensity levels would have to be assigned to the same brightness level. While the distinction of subtle changes in the perception of photopic phenomena was crucial for our study, we considered that 16 levels were optimal and most reliable owing to the discretization of the brightness measurement scale.

**Table 1:** Levels of probe diode brightness.

Level	Luminous intensity (mcd)	Relative deviation from fitted value because of quantized dynamics of diodes
0	0.00	—
1	0.37	-10%
2	1.32	+24%
3	2.50	+18%
4	3.75	-0.94%
5	5.03	-22%
6	12.3	+14%
7	20.2	+14%
8	28.1	-2.8%
9	45.8	+2.0%
10	76.1	+1.1%
11	118	-2.7%
12	192	-1.6%
13	308	-1.7%
14	500	-0.57%
15	801	-0.69%
16	1300	+0.44%

### Dysphotopsia measurements and pPSF

**Fig 6:**
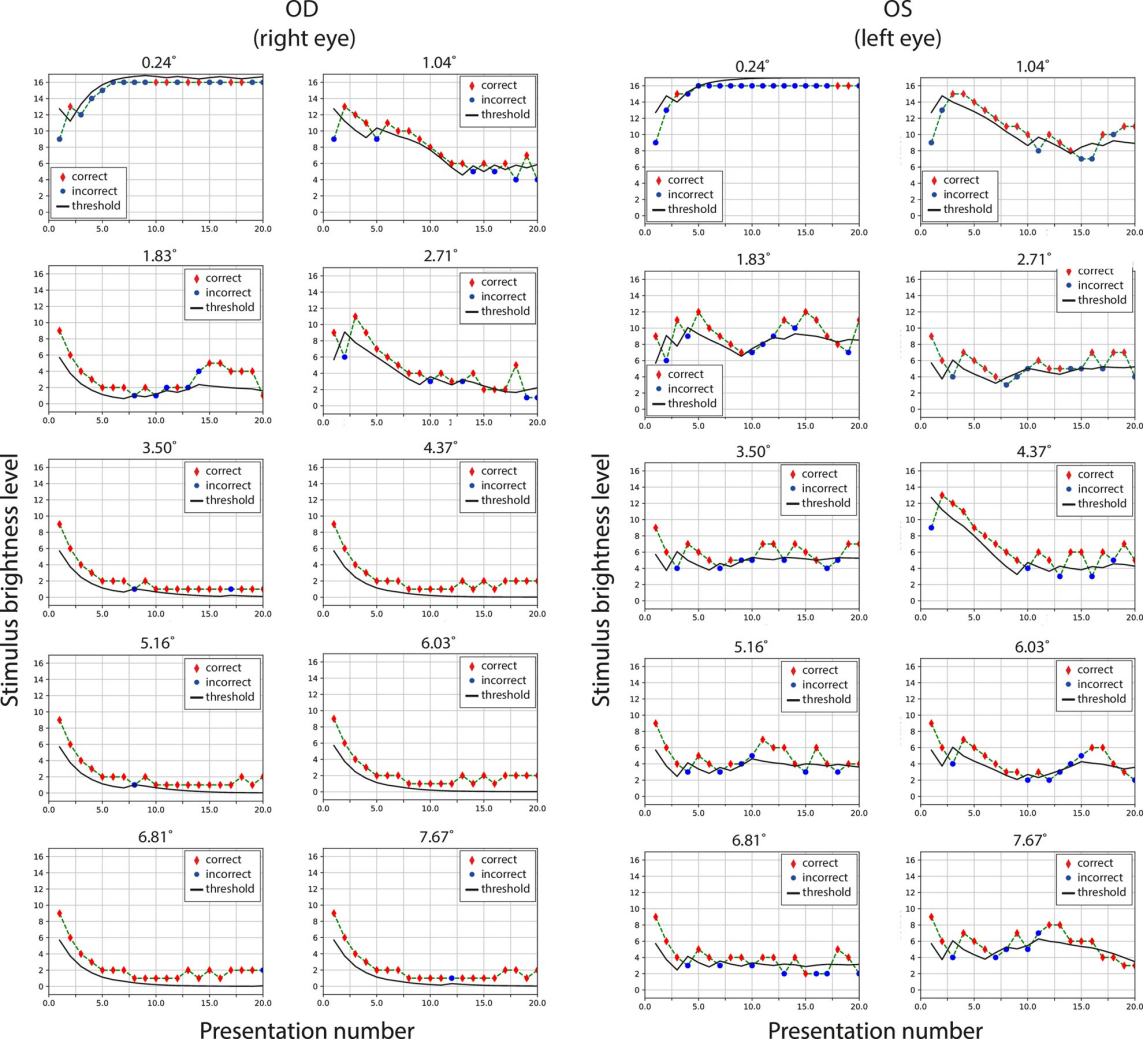
Courses of all tests of the LYS patient. Subsequent stimulus brightness levels for each trial were calculated using the PSI algorithm. Red diamonds indicate the correct answers, whereas blue dots indicate the incorrect answers. The black line defines the threshold (α parameter) adjustment after each trial as the test progresses. The right eye was implanted with an AcrySof IQ Vivity IOL. The left eye was affected by a cataract. Plot titles indicate the angular distance from the central light spot (as a parameter of a given test) that induced dysphotopsia.

Herein, we discuss the detailed results of one selected case (LYS patient). The left eye had not been operated on (despite the cataract signs), and in the right eye, AcrySof IQ Vivity IOL was implanted (see details in the Material and methods section). Her case is representative of our study, and it was possible to apply the proposed method to the analysis of cataract blurring and IOL dysphopsia in one subject. Fig 6 presents the course of individual trials conducted in a random order according to the algorithm presented in Fig 4. The vertical axis shows the intensity of the stimulus, that is, the brightness of the probe diode, and the horizontal axis shows successive trials. Red diamonds indicate correct responses of LYS, whereas blue dots indicate incorrect answers. The black line defines the threshold estimation after each trial.

**Fig 7:**
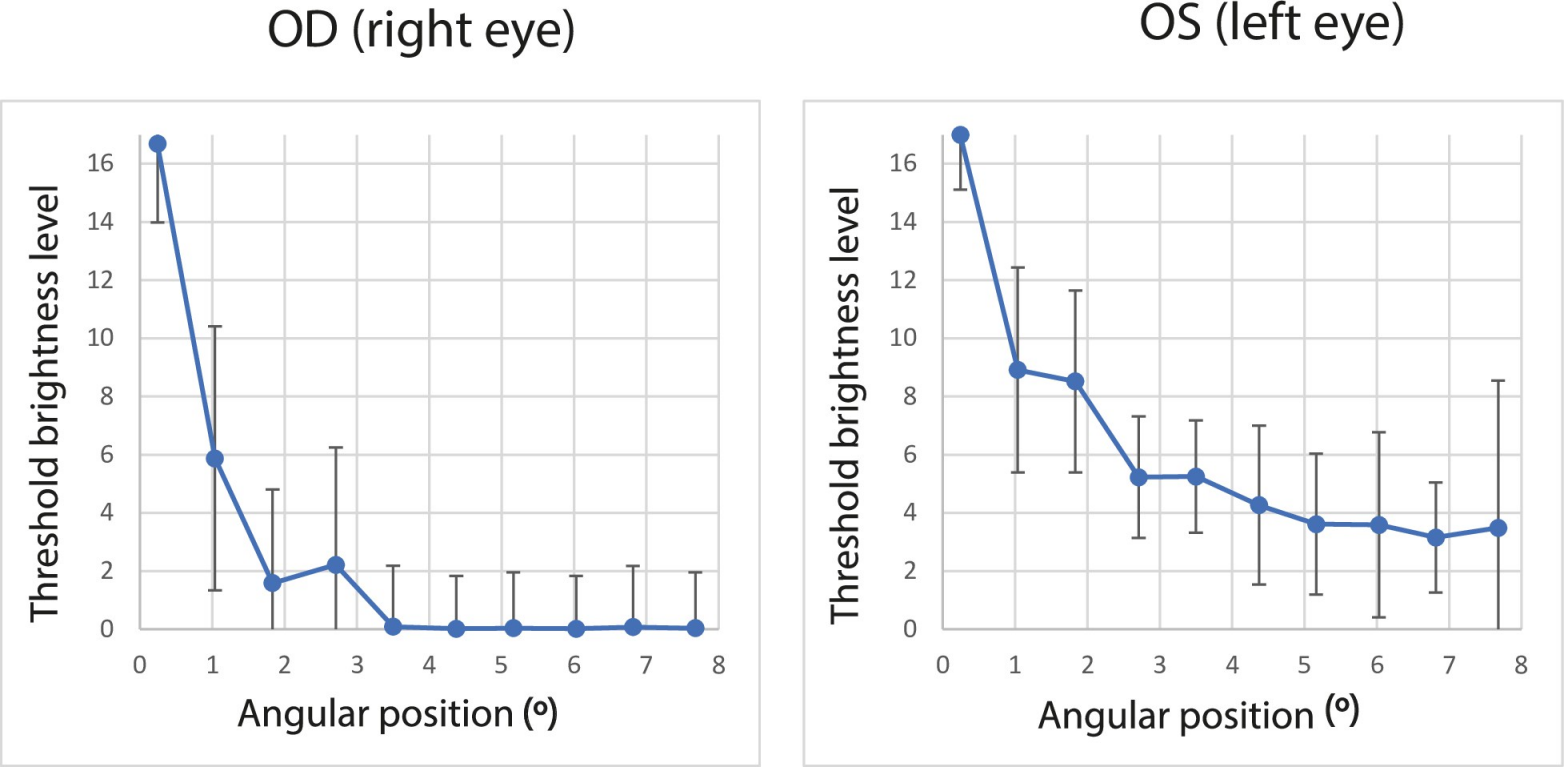
Perceptual brightness of positive dysphotopsia as a function of the angular distance of the central light spot for the LYS patient. The right eye was implanted with AcrySof IQ Vivity IOL. The left eye was affected by a cataract. The uncertainty error is the confidence interval of 95%.

The probability P of the correct answer in each trial can be modeled by the logistic psychometric function [29],

P(x;α,β,γ,λ)=γ+(1−λ−γ)F(x;α,β), (2)

with

$$F(x;\alpha ,\beta )=\frac{1}{1+exp[-\beta (x-\alpha )]}$$ (3)

where the guess rate was estimated to be γ = 0.17 (one in six possible stimulus directions) and the lapse rate λ was assumed to be zero. The parameter α corresponds to the threshold stimulus level, where the probability of a correct answer is 50%, while β is the function’s slope at this point. Both were fitted after each trial (the current value of α is marked by black a line in Fig 6) and used in the PSI adaptive procedure to determine the stimulus value for the subsequent stimulus level. After N = 20 trials, the final value of α was interpreted as the just-noticeable stimulus brightness at a given angular position in the visual field (see dots in Fig 7). This corresponds to the subjective positive dysphotopsia intensity as a function of distance from the central light spot.

The final parameter β was used to determine the level of uncertainty and the brightness levels were calculated based on the probabilities of correct answers of 2.5% and 97.5%, i.e., with a confidence interval of 95% (see error bars in Fig 7).

**Fig 8:**
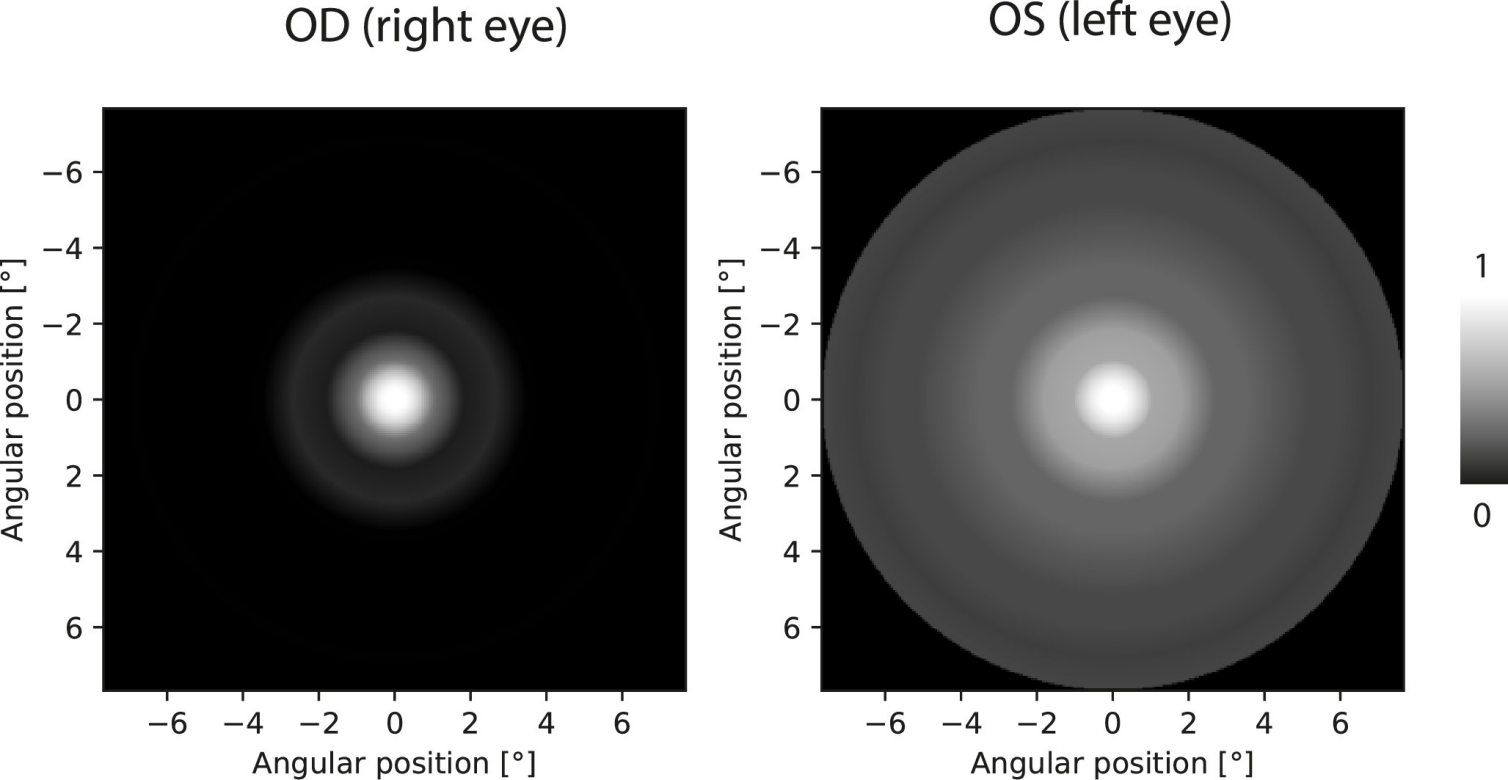
Map of perceptual PSF for the LYS patient based on data presented in Fig 7. The right eye was implanted with an AcrySof IQ Vivity IOL. The left eye was affected by a cataract. The axes are scaled in angular degrees of the visual field, and the center point is related to the position of the central light spot.

**Fig 9:**
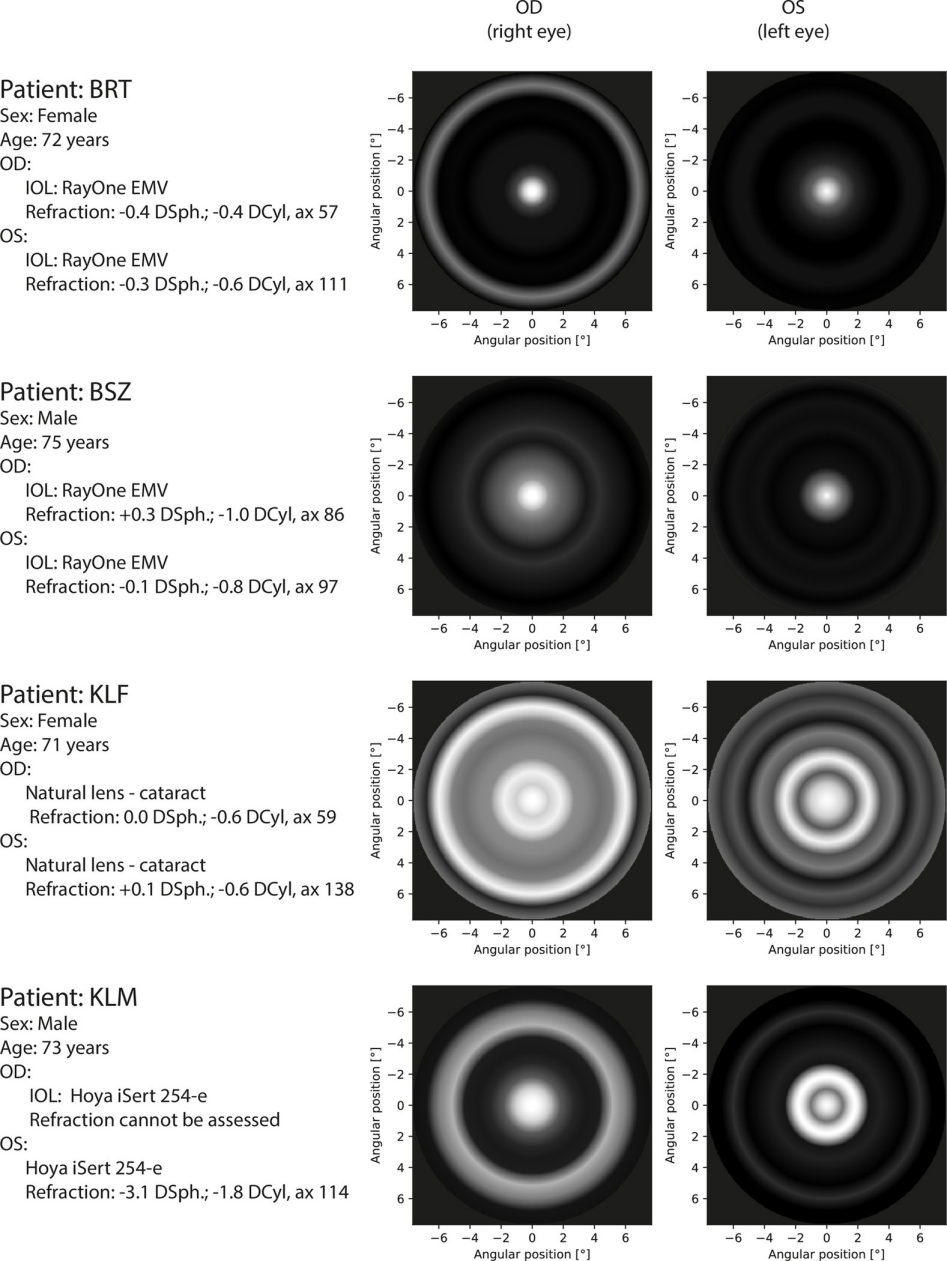
Perceptual PSF for first part of patients. All eyes were measured with corrected distance visual acuity.

**Fig 10:**
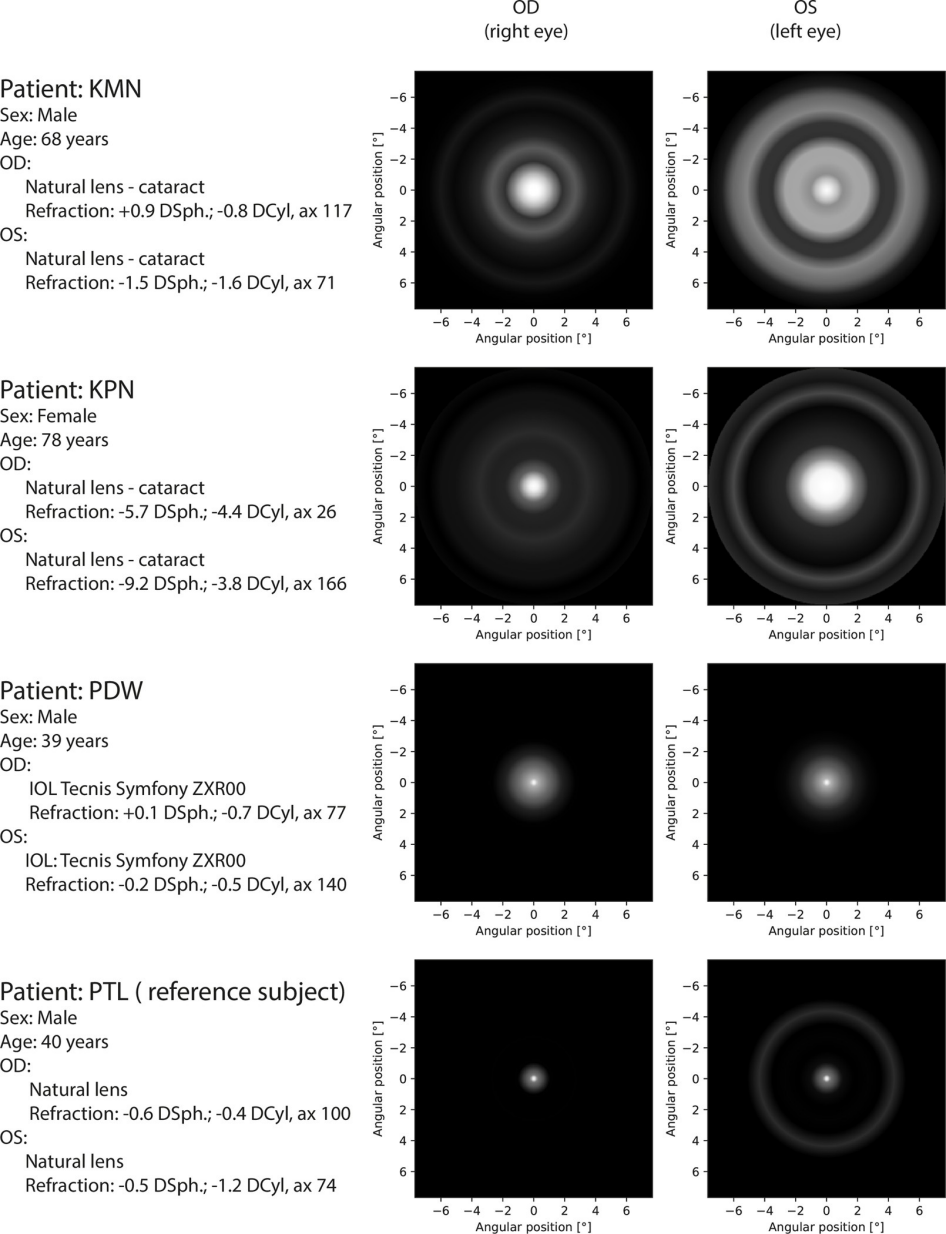
Perceptual PSF for second part of patients. All eyes were measured with corrected distance visual acuity.

**Fig 11:**
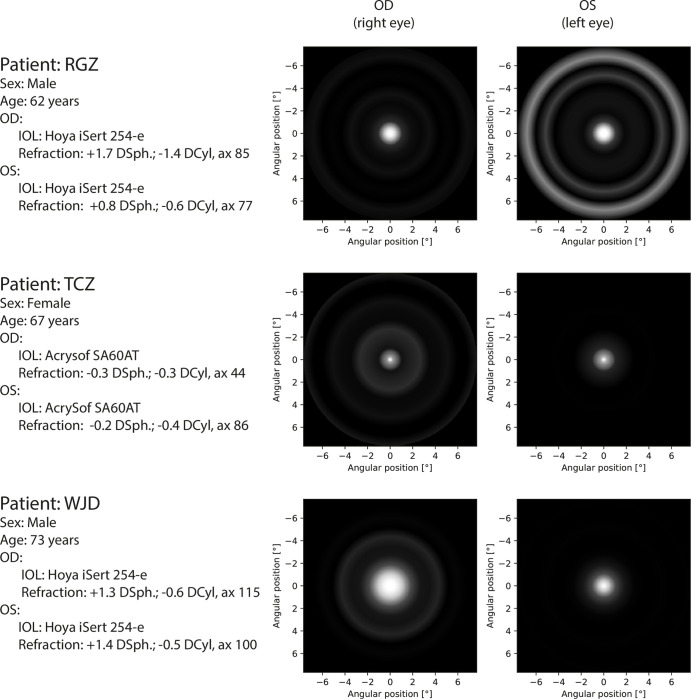
Perceptual PSF for third part of patients. All eyes were measured with corrected distance visual acuity.

The determined photopsia intensity is presented in Fig 7 as a function of the distance from the central light spot (assuming rotational symmetry). Fig 8 corresponds to the perceptual PSF and represents an 8-bit grayscale map (256 levels). The values between the measured stimuli were predicted using spline interpolation in the pPSF images presented in Figs 8–11. Both presentations have axes scaled in degrees of angular distance from the central light spot to induce photopsia.

The measured pPSFs for all patients are presented in Figs 9–11. According to the grayscale introduced by the calibration procedure, the brightness in the pPSF maps represents the illusory light in the patient’s visual field. The expected occurrence of the halo effect was visible in several patients. Nevertheless, it was also partially present, even within the natural crystalline lens of the youngest healthy patient (PTL). In all cases, vision was characterized based on glare; however, PTL experienced the least prominent effects.

Images of pPSF for subjects with cataracts (KLF, KMN) show many rings with different thicknesses and brightness levels. Although the origins of these rings are partially attributed to the measurement uncertainty, it is shown that crystalline lens opacity is a serious problem for the vision of these patients as it creates huge and heterogeneous glare. By contrast, the spatial extent of the cataract in KPN’s eyes was diagnosed to be smaller than those in KLF and KMN but was still accompanied by a large glare without significant halos. Such findings are in line with the patients’ self-perceptions described during the preliminary assessments of their eyes.

Patients with IOLs perceive some glare and low-intensity halo effects. Because both the transmission of the different IOLs and aberrations of each of the patients’ eyes are not precisely known, these effects may be related to the type of the IOL, individual eye aberration profile, as well as neural perception properties. TCZ had monofocal AcrySof SA60AT implanted in both eyes and the pPSF was similar to the natural lens case (PTL) with insignificantly broader glare effects.

Other results were obtained for monofocal Hoya iSert IOLs. Patients with this solution (RGZ, WJD) manifest noticeable strong glare or even weak halo effects in their pPSFs. A very poor response was associated with KLM (who also used Hoya iSert) who reported “rays and bursts” in his vision during his initial interview. Owing to problems in autorefractometry (right eye measurements were impossible), a fundoscopy was performed. This subject needed reimplantation of the IOL because of the asymmetrical and opaque position of the implant. His problems with vision were obvious in pPSFs; these were similar to cataract patterns.

Perpetual PSFs of patients with multifocal or EDOF IOLs (BRT, BSZ, PDW) are visibly characterized by increased amounts of glare because of the complicated lens optics. In some cases, low-intensity halos were found. It can be noticed that the quality of vision by TECNIS Symfony (PDW) is better than that for RayOne EMV (BRT, BSZ), for which additional rings were obtained. The TECNIS Symfony advantage is in line with the findings in [32].

In the interviews, all patients with IOLs (except KLM) did not indicate any noticeable problems with vision. This may be attributed to self-reference to the pre-surgical, cataract vision states. Nevertheless, it is worth noting that in each tested case, the objective representations of the subjective visions were different in the left and right eyes. This shows the sensitivity of the proposed method both to the type of intraocular lenses used and to visual defects.

## Conclusions

In this work, we presented a method of measuring dysphosia in real eyes through a perceptual measurement of the positive dysphotopsia caused in night vision by a point-like light source placed in the center of the field of vision. Although analysis of the factors influencing dysphosia was beyond the scope of this work, it is worth emphasizing that the spectral properties of the implanted IOLs and the light sources used, as well as the mood and fatigue of the subjects may influence the size and intensity of dysphotopsias perceived. Blue light turned out to be particularly important for such effects, and appropriate filters that cut it off can reduce dysphopsia and generally affect visual comfort [33–38]. Moreover, the pupillary response immediately after turning on of the central diode is triggered by the M1 ganglion cells through the olivary pretectal nucleus. These neurons have their peak sensitivity at 490 nm what can affect subjects’ effective light sensitivity [39]. In the future, it is worth examining different LED light sources with higher or lower proportion of blue near the wavelength of 490 nm.

The visual point spread function was discussed in the literature [40–42]. The important conclusion drawn based on the studies in the literature is the classification of the PSF responses to the foveal (<1°) and stray-light (>1°) fields. The former will impact VA, while the latter will involve contrast sensitivity for larger-scale objects and the visual scene as a whole [42]. In our study, pPSF was dedicated to the description of glare and halo effects and could not be used to interpret VA-linked characteristics.

To perform examinations according to the proposed method, we constructed a patented device for central vision far-field perimetry. This device has a point-like, strong phosphor-based white diode in the center of the field of view, and a series of probe diodes located radially from 0.24° to 7.67° away from it, at a distance of 5 m in front of the patient’s eyes. During the test, a minimal, just-noticeable luminous intensity could be measured at any point in the visual field, which allowed us to draw an illusory light distribution. With simple interpolation and assuming rotational symmetry, we constructed two-dimensional grayscale images analogous to the optical point spread function, wherein the output light distribution was also recorded as an image of a point light source in the input plane.

Twenty-four eyes were studied, including the post-operative study of different IOL corrections, healthy natural lenses, and natural lenses with cataracts. Dysphotopsia can be unequivocally recognized as a condition related to the aging of the eye and the implanted IOL. In this study, the healthy young patient (PTL) experienced minimal halos and glares, whereas older people with cataracts, or even IOLs, were affected more severely. Results also showed evident differences between monofocal (RGZ, TCZ) and EDOF/multifocal (BRT, PDW) lens implants attributed to the complexity of the lens optics. The proposed method of night vision assessment can also provide information about the quality of the surgical procedures describing the real distribution of light perceived by the patient (KLM).

The duration of measurements performed in this study did not exceed 15 min, which could affect the error estimation and resolution (Fig 7). In clinical applications, the time of measurement should consider both: patient comfort and expected precision of dysphotopsia assessment (Figs 9–11).

It is difficult to interpret our results in the context of the existing literature because of differences in the experimental protocols (central versus peripheral source of light) and estimation procedures. Most of the studies refer to halo size determinations in monofocal and multifocal IOLs used in cataract correction [43] and to the assessment of the diagnostic capacity [21]. In [40], authors using a vision monitor (MonCv3, Metrovision) reported larger halo radius in multifocal (range 165 to 297 arcmin) than in monofocal (range 99 to 286 arcmin) groups (minimum 18 subjects per sample). Another report discusses halo effects associated with IOLs by an MTF bench-based system and by the high-dynamic range (HDR) image photometer-based system [14]. In the last solution, two visualizations were proposed, namely halo luminance profiles and two-dimensional images representing halo data. The HDR system was shown to be realistic in the simulation of night-driving conditions and corresponded to patients’ subjective perceptions. Our results were in line with these findings, thus showing similarities in halo perception reconstruction (qualitatively in the form of grayscale images) and quantitatively considering the type IOLs of vision correction. Our study extended beyond the existing reports and discussed the analyzed healthy eye cases. The proposed analysis confirmed that there are many additional sources of disphotopsias occurrences not only related to implants as the optical system of the healthy eye may also exhibit photic effects. The great potential of this approach relies on its capacity to describe imaging properties of optical systems. This approach opens a way to precisely parametrize perceived photopsia based on aberration characterization. Our solution may be helpful in the construction of optical phase masks by creating the same pattern of PSF as that of the pPSF of a specific patient’s eye. In this way, the proposed solution could study the vision of the tested eye; physicians will not only be able to see what the patient sees, but they also will study the advantages and disadvantages of possible eye correction methods. However, this application requires the knowledge of correlations between IOL optical properties (i.e., their PSFs) and perceptual characteristics (i.e., the pPSFs), which is an aspect of the scope of future research.

Finally, our approach can also open new possibilities for IOL and contact lens designers. Their new products may be evaluated quasi-perceptually at the optical bench by applying pPSF phase masks like the perceptual aberration profile of the actual human eye. This possibility becomes a first step to personalized vision correction.

## Supporting information

S1 File Detailed graphs of all trials in this study with a legend. (PDF)

S2 File All data presented in the manuscript with a legend. (ZIP)

## References

[1] Das S, Sun X, Dadashova B, Rahman MA, Sun M. Identifying patterns of key factors in sun glare- related traffic crashes. Transp. Res. Rec. 2022;2676 (2):165–175. doi: 10.1177/0361198121103789

[2] Masket S, Fram NR. Pseudophakic dysphotopsia: review of incidence, cause, and treatment of positive and negative dysphotopsia. Ophthalmology. 2021;128(11):e195–e205. doi: 10.1016/j.ophtha.2020.08.009 32800744

[3] Hu J, Sella R, Afshari NA. Dysphotopsia: a multifaceted optic phenomenon. Curr. Opin Ophthalmol. 2018;29(1):61–68. doi: 10.1097/ICU.0000000000000447 29084005

[4] Tester R, Pace NL, Samore M, Olson RJ. Dysphotopsia in phakic and pseudophakic patients: incidence and relation to intraocular lens type. J. Cataract Refract. Surg. 2000;26(6):810–816. doi: 10.1016/S0886-3350(00)00427-2 10889424

[5] Dick HB, Krummenauer F, Schwenn O, Krist R, Pfeiffer N. Objective and subjective evaluation of photic phenomena after monofocal and multifocal intraocular lens implantation. Ophthalmology. 1999;106(10):1878–1886. doi: 10.1016/S0161-6420(99)90396-2 10519580

[6] Alba-Bueno F, Garzón N, Vega F, Poyales F, Millán MS. Patient-perceived and laboratory-measured halos associated with diffractive bifocal and trifocal intraocular lenses. Curr. Eye Res. 2018;43(1):35–42. doi: 10.1080/02713683.2017.1379541 29161162

[7] Castro-Torres JJ, Martino F, Casares-López M, Ortiz-Peregrina S, Ortiz C. Visual performance after the deterioration of retinal image quality: induced forward scattering using Bangerter foils and fog filters. Biomed. Opt. Express. 2021;12(5):2902–2918. doi: 10.1364/BOE.424715 34123509 PMC8176796

[8] Mencucci R, Cennamo M, Venturi D, Vignapiano R, Favuzza E. Visual outcome, optical quality, and patient satisfaction with a new monofocal IOL, enhanced for intermediate vision: preliminary results. J. Cataract Refract. Surg. 2020;46(3):378–387. doi: 10.1097/j.jcrs.0000000000000061 32050218

[9] Rodov L, Reitblat O, Levy A, Assia EI, Kleinmann G. Visual outcomes and patient satisfaction for trifocal, extended depth of focus and monofocal intraocular lenses. J. Refract. Surg. 2019;35(7):434–440. doi: 10.3928/1081597X-20190618-01 31298723

[10] Breyer DH, Kaymak H, Ax T, Kretz FTA, Auffarth GU, Hagen PR. Multifocal intraocular lenses and extended depth of focus intraocular lenses. Asia Pac. J. Ophthalmol. (Phila) 2017;6(4):339–349. doi: 10.22608/APO.2017186 28780781

[11] Petelczyc K, Kolodziejczyk A, Błocki N, Byszewska A, Jaroszewicz Z, Kakarenko K, et al. Model of the light sword intraocular lens: in-vitro comparative studies. Biomed. Opt. Express 2020;11(1);40–54. doi: 10.1364/BOE.11.000040 32010498 PMC6968750

[12] Palomino-Bautista C, Sánchez-Jean R, Carmona-González D, Piñero DP, Molina-Martin A. Subjective and objective depth of field measures in pseudophakic eyes: comparison between extended depth of focus, trifocal and bifocal intraocular lenses. Int. Ophthalmol. 2020;40(2):351–359. doi: 10.1007/s10792-019-01186-6 31583551

[13] Vega F, Alba-Bueno F, Millán MS, Varón C, Gil MA, Buil JA. Halo and through-focus performance of four diffractive multifocal intraocular lenses. Invest. Ophthalmol. Vis. Sci. 2015;56(6):3967–3975. doi: 10.1167/iovs.15-16600 26098463

[14] Carson D, Lee S, Alexander E, Wei X, Lee S. Comparison of two laboratory-based systems for evaluation of halos in intraocular lenses. Clin. Ophthalmol. 2018;12:385–393. doi: 10.2147/OPTH.S152201 29503526 PMC5826305

[15] Williams DR, Brainard DH, McMahon MJ, Navarro R. Double-pass and interferometric measures of the optical quality of the eye. JOSA A, 1994;11(12): 3123–3135. doi: 10.1364/josaa.11.003123 7837000

[16] Ginis H, Sahin O, Pennos A, Artal P. Compact optical integration instrument to measure intraocular straylight. Biomed. Opt. Ex. 2014;5(9):3036–3041. doi: 10.1364/BOE.5.003036 25401017 PMC4230852

[17] Savini G, Schiano-Lomoriello D, Balducci N, Barboni P. Visual performance of a new extended depth-of-focus intraocular lens compared to a distance-dominant diffractive multifocal intraocular lens. J. Refract. Surg. 2018;34(4):228–235. doi: 10.3928/1081597X-20180125-01 29634837

[18] Elliott MA, Nothelfer C, Xiong C, Szafir DA. A design space of vision science methods for visualization research. IEEE Trans. Vis. Comput. Graph. 2021;27(2):1117–1127. doi: 10.1109/TVCG.2020.3029413 33090954

[19] Ungewiss J, Schiefer U, Eichinger P, Crabb DP, Jones PR. Does intraocular straylight predict night driving visual performance? Correlations between straylight levels and contrast sensitivity, halo size, and hazard recognition distance with and without glare. Front. Hum. Neurosci. 2022;16:910620. doi: 10.3389/fnhum.2022.910620 36177386 PMC9514855

[20] Puell MC, Pérez-Carrasco MJ, Barrio A, Palomo-Alvarez C. Normal values for the size of a halo produced by a glare source. J. Refract. Surg. 2013;29(9):618–622. doi: 10.3928/1081597X-20130819-03 24016347

[21] Palomo-Álvarez C, Puell MC. Capacity of straylight and disk halo size to diagnose cataract. J. Cataract. Refract. Surg. 2015;41(10):2069–2074. doi: 10.1016/j.jcrs.2015.10.047 26703281

[22] Johnson CA, Wall M, Thompson HS. A history of perimetry and visual field testing. Optom. Vis. Sci. 2011;88(1):E8–E15. doi: 10.1097/OPX.0b013e3182004c3b 21131878

[23] Hoya Surgical Optics, “iSert Specs Sheet”, https://www.hoyasurgicaloptics.com/isert/iol.

[24] MyAlconProfessionals, “AcrySof® IQ Monofocal and AcrySof® IQ Toric IOLs,” https://www.myalcon.com/uk/professional/cataract-surgery/iols/acrysof-iq-monofocal/.

[25] Rayner, “Extending range without compromise”, https://rayner.com/en/iol/rayone-emv/.

[26] MyAlconProfessionals, “AcrySof IQ Vivity® IOL,” https://www.myalcon.com/international/professional/cataract-surgery/iols/vivity/.

[27] TECNIS Symfony® Extended Range of Vision IOL, Product Leaflet Z311215 Rev. 01.

[28] Hsia Y., Graham C. H. Spectral sensitivity of the cones in the dark adapted human eye. Proc Natl Acad Sci U S A, 1952;38(1), 80–85. doi: 10.1073/pnas.38.1.80 16589059 PMC1063505

[29] Kingdom FAA, Prins N, Psychophysics. A Practical Introduction (Elsevier Ltd., 2010).

[30] Luce RD, Edwards W. The derivation of subjective scales from just noticeable differences. Psychol. Rev. 1958;65(4):222–237. doi: 10.1037/h0039821 13579090

[31] Kontsevich LL, Tyler CW. Bayesian adaptive estimation of psychometric slope and threshold. Vision Res. 1999;39(16):2729–2737. doi: 10.1016/s0042-6989(98)00285-5 10492833

[32] Rampat R, Gatinel D. Multifocal and extended depth-of-focus intraocular lenses in 2020. Ophthalmology. 2021;128(11):e164–e185. doi: 10.1016/j.ophtha.2020.09.026 32980397

[33] Hammond B. R., Gardner C. R., Renzi-Hammond L. The Effects of Blue-Light Filtering Intraocular Implants on Glare Geometry. Curr. Eye Res, 2023;48(7), 639–644. doi: 10.1080/02713683.2023.2192446 37074213

[34] Gray R., Perkins S. A., Suryakumar R., Neuman B., Maxwell W. A. Reduced effect of glare disability on driving performance in patients with blue light–filtering intraocular lenses. J Cataract Refract Surg, 2011;37(1), 38–44, doi: 10.1016/j.jcrs.2010.07.034 21183098

[35] Gray R., Hill W., Neuman B., Houtman D., Potvin R. Effects of a blue light–filtering intraocular lens on driving safety in glare conditions. J Cataract Refract Surg, 2012;38(5), 816–822, doi: 10.1016/j.jcrs.2011.11.047 22520305

[36] Baeza-Moyano D., Arranz-Paraíso D., Sola Y., González-Lezcano R. A. Suitability of blue light filters for eye care. Eur. Phys. J. Plus, 2022;137(7), 817. doi: 10.1140/epjp/s13360-022-03045-3

[37] Ouyang X. I. N. L. I., Yang J., Hong Z., Wu Y., Xie Y., Wang G. Mechanisms of blue light-induced eye hazard and protective measures: a review. Biomed Pharmacother, 2020;130, 110577, doi: 10.1016/j.biopha.2020.110577 32763817

[38] Popov I., Jurenova D., Valaskova J., Sanchez-Chicharro D., Stefanickova J., Waczulikova I., et al. Effect of blue light filtering intraocular lenses on visual perception. Medicina, 2021;57(6), 559., doi: 10.3390/medicina57060559 34206059 PMC8226562

[39] Schroeder M. M., Harrison K. R., Jaeckel E. R., Berger H. N., Zhao X., Flannery M. P., et al. The roles of rods, cones, and melanopsin in photoresponses of M4 intrinsically photosensitive retinal ganglion cells (ipRGCs) and optokinetic visual behavior. Front. Cell. Neurosci., 2018;12, 203., doi: 10.3389/fncel.2018.00203 30050414 PMC6052130

[40] Ginis H, Pérez GM, Bueno JM, Artal P. The wide-angle point spread function of the human eye reconstructed by a new optical method. J. Vis. 2012;12(3):20. doi: 10.1167/12.3.20 22451158

[41] Watson AB. Computing human optical point spread functions. J. Vis. 2015;15(2):26. doi: 10.1167/15.2.26 25724191

[42] van den Berg TJTP. The (lack of) relation between straylight and visual acuity. Two domains of the point‐spread‐function. Ophthalmic Physiol Opt. 2017;37(3):333–341. doi: 10.1111/opo.12368 28271538

[43] Puell MC, Pérez-Carrasco MJ, Hurtado-Ceña FJ, Álvarez-Rementería L. Disk halo size measured in individuals with monofocal versus diffractive multifocal intraocular lenses. J. Cataract. Refract. Surg. 2015;41(11):2417–2423. doi: 10.1016/j.jcrs.2015.04.030 26703491

