# Supplementary material for: Use of the perceptual point-spread function to assess dysphotopsias
Source: PLoS One. 2024 Jul 19;19(7):e0306331. doi: 10.1371/journal.pone.0306331 (PMC11259305; doi:10.1371/journal.pone.0306331)
Supplement: S1 File — (PDF) [file pone.0306331.s001.pdf]

S1_File.pdf

Supporting Information to PLOS ONE publication
Use of the perceptual point-spread function to assess dysphotopsias

This document (S1_File.pdf) contains detailed graphs of all trials in this study. For each patient and each of the patient's eyes presented in Figs. 9-11 in the manuscript, there is a graph presenting the course of individual trials for each angular position of the test stimulus, analogous to Fig. 6 in the manuscript (with axis labels the same as in Fig. 6). Additionally, there is a graph presenting each final subjective dysphotopsia intensity as a function of distance from the central light spot, analogous to Fig. 7 in the manuscript.

Contents

1 BRT **2**

2 BSZ **3**

3 KLF **4**

4 KLM **5**

5 KMN **6**

6 KPN **7**

7 LYS **8**

8 PDW **9**

9 PTL **10**

10 RGZ **11**

11 TCZ **12**

12 WJD **13**

1 BRT

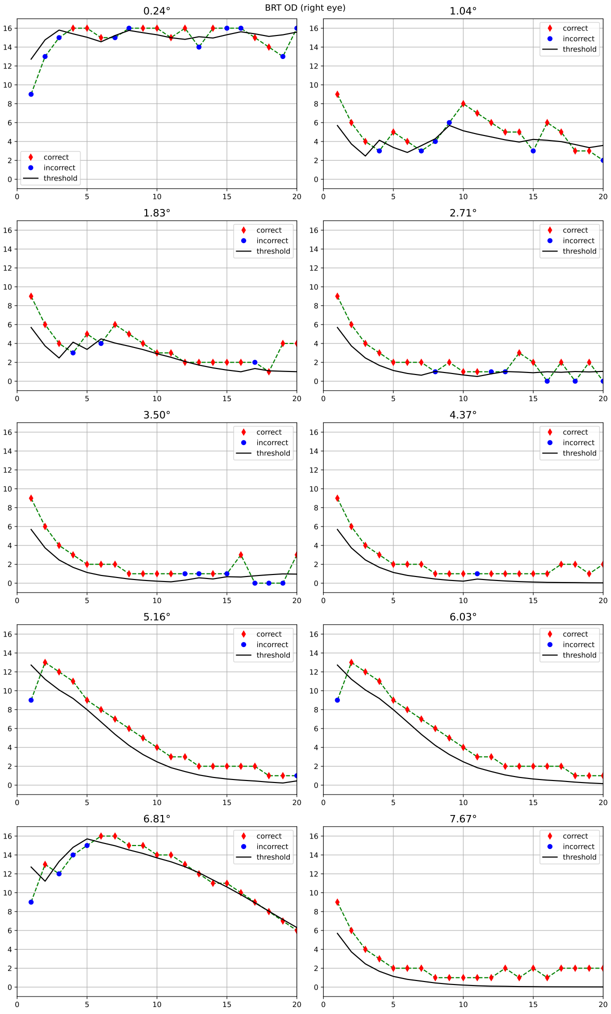

(a) OD (right eye)

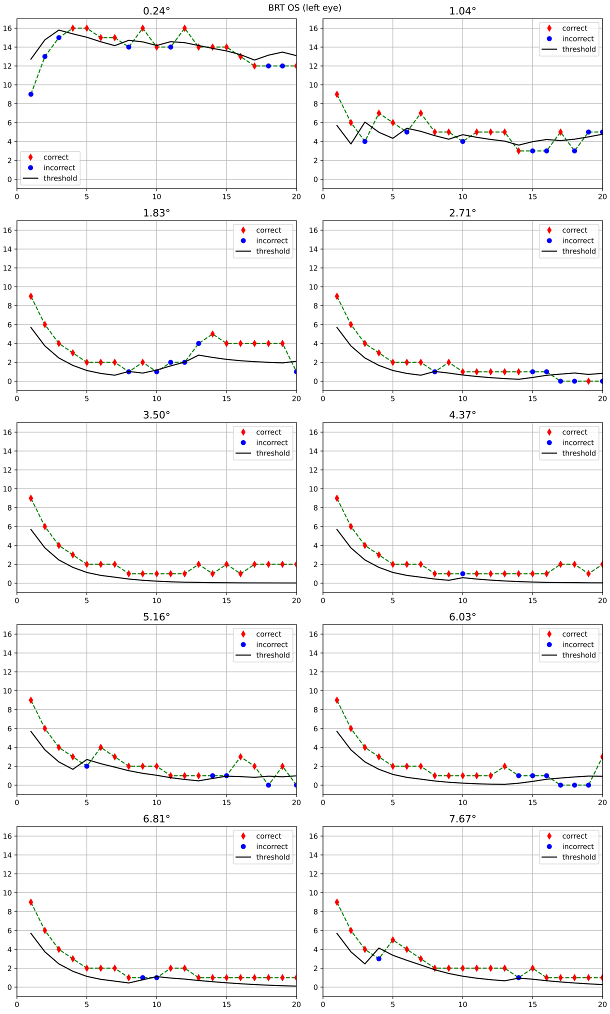

(b) OS (left eye)

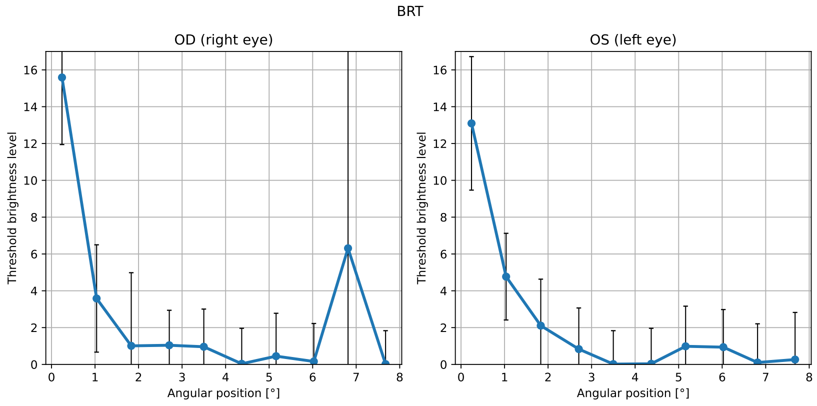

(c) radial plot

2 BSZ

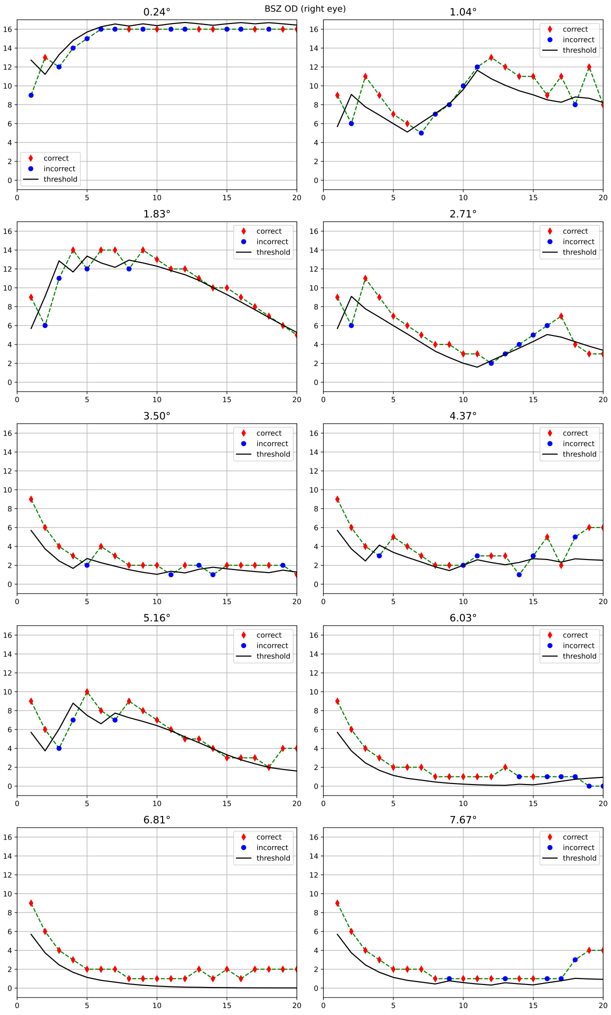

(a) OD (right eye)

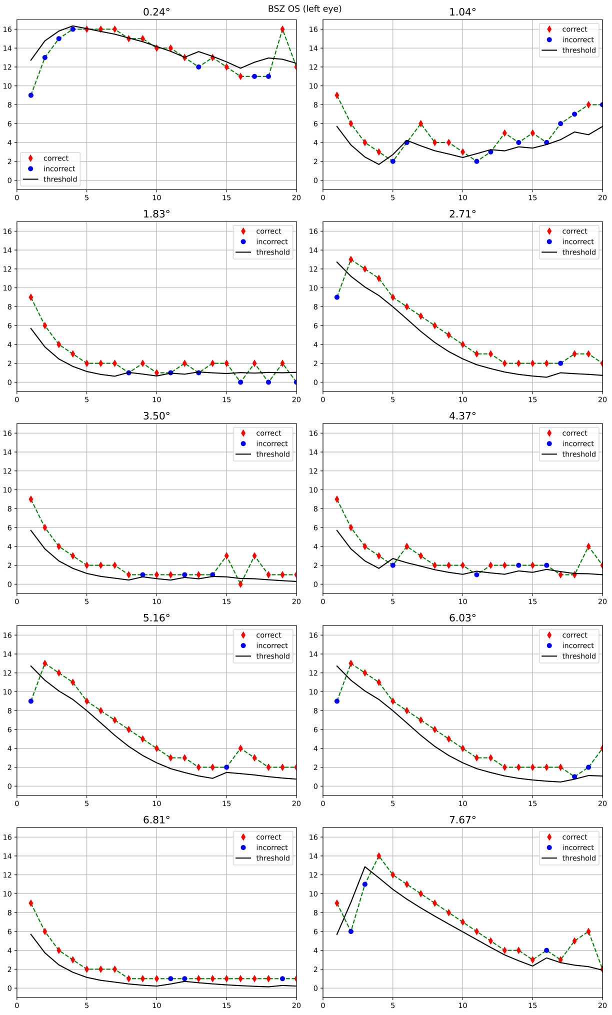

(b) OS (left eye)

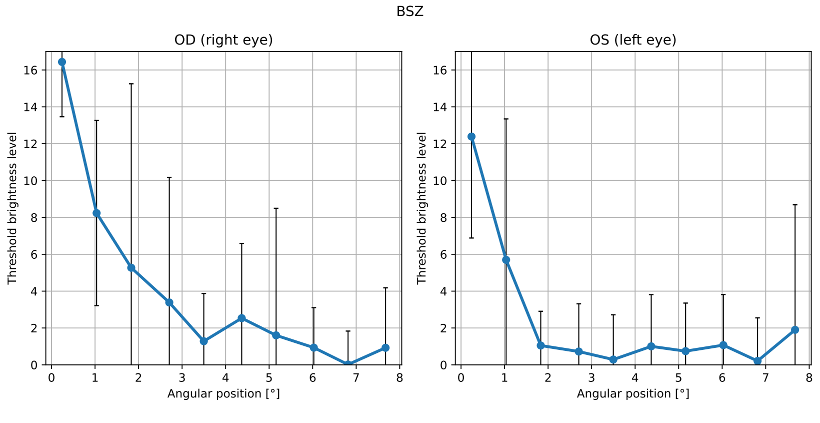

(c) radial plot

3 KLF

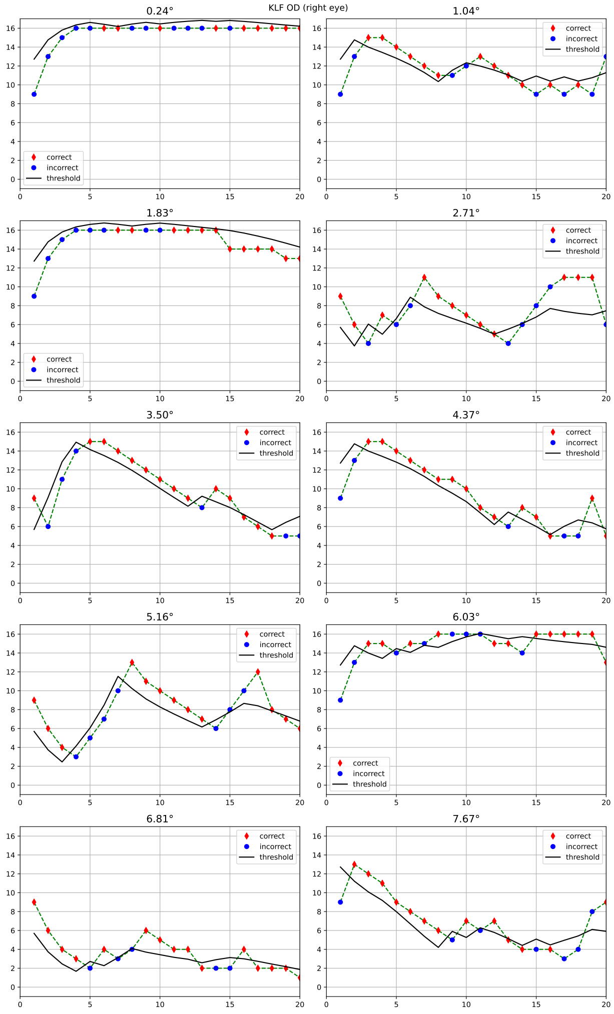

(a) OD (right eye)

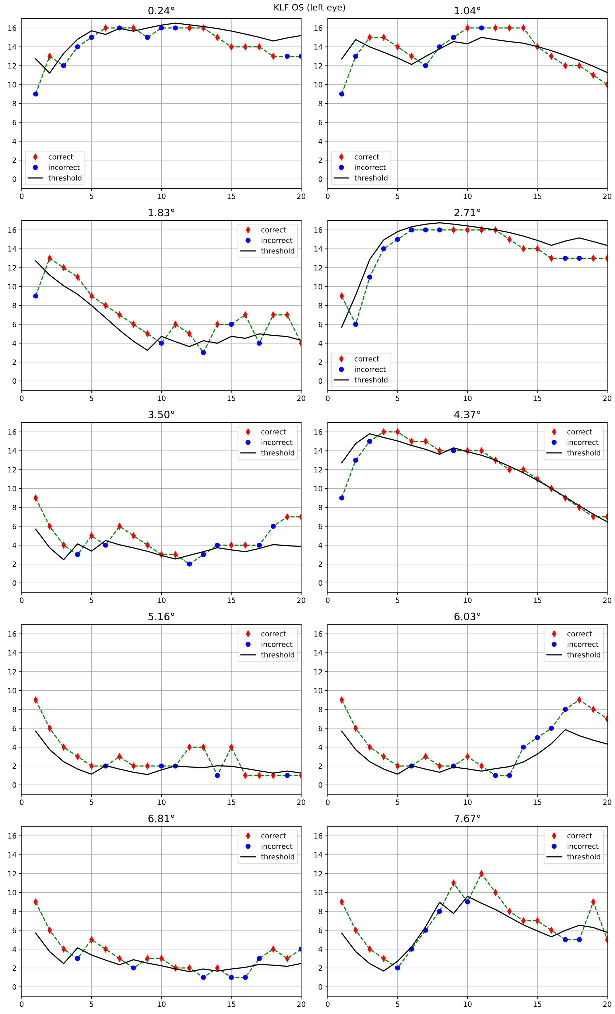

(b) OS (left eye)

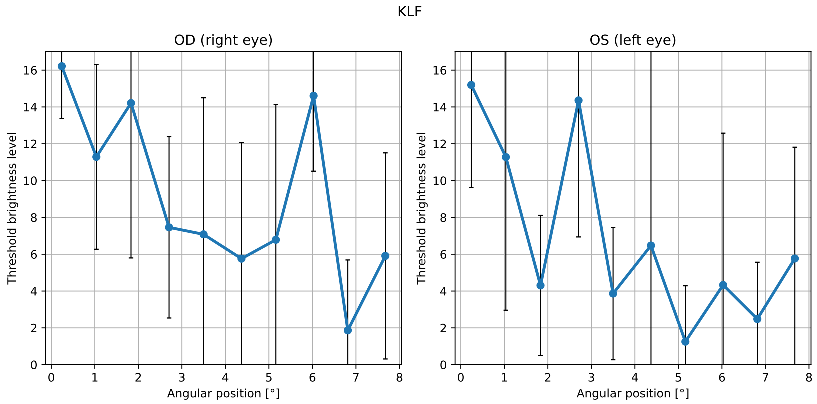

(c) radial plot

4 KLM

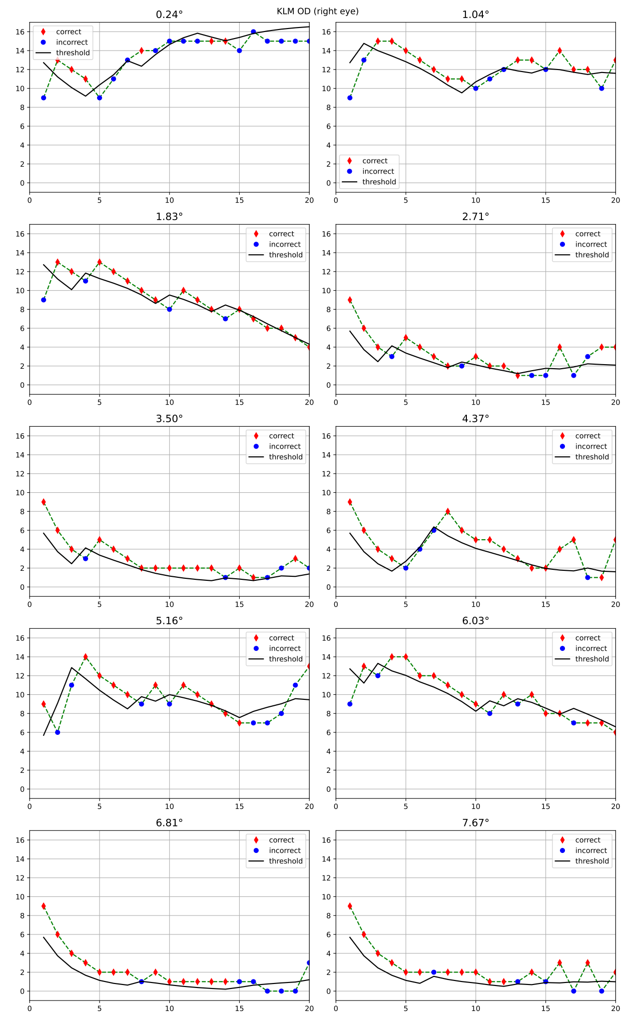

(a) OD (right eye)

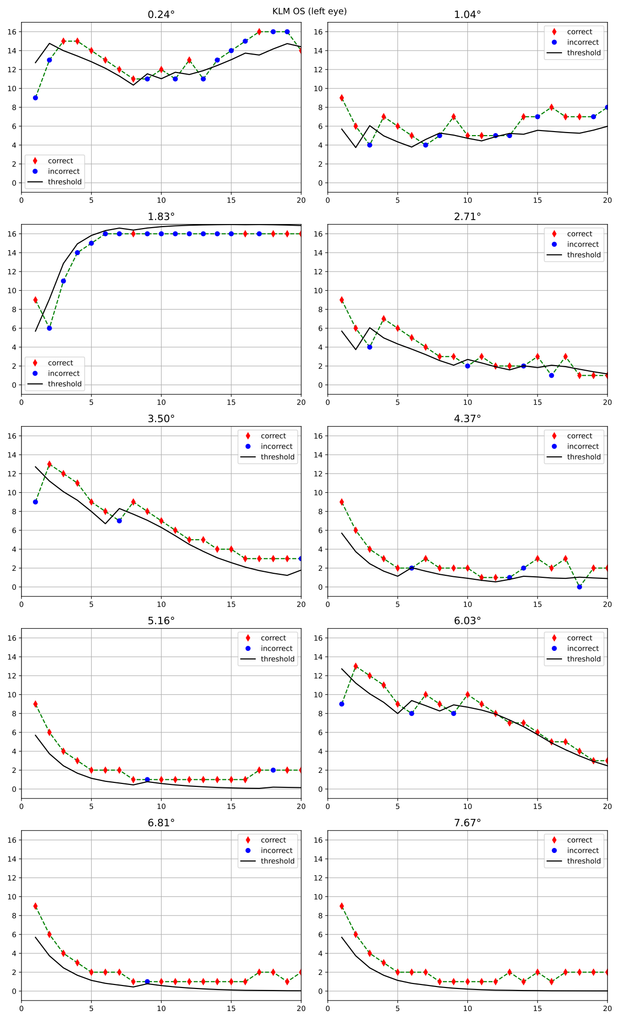

(b) OS (left eye)

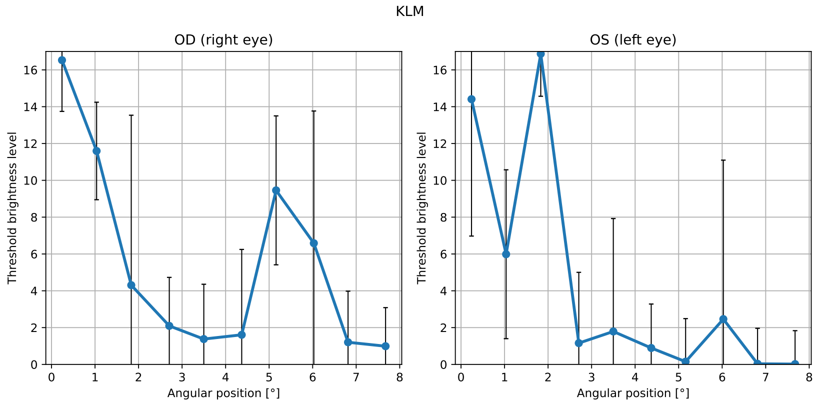

(c) radial plot

5 KMN

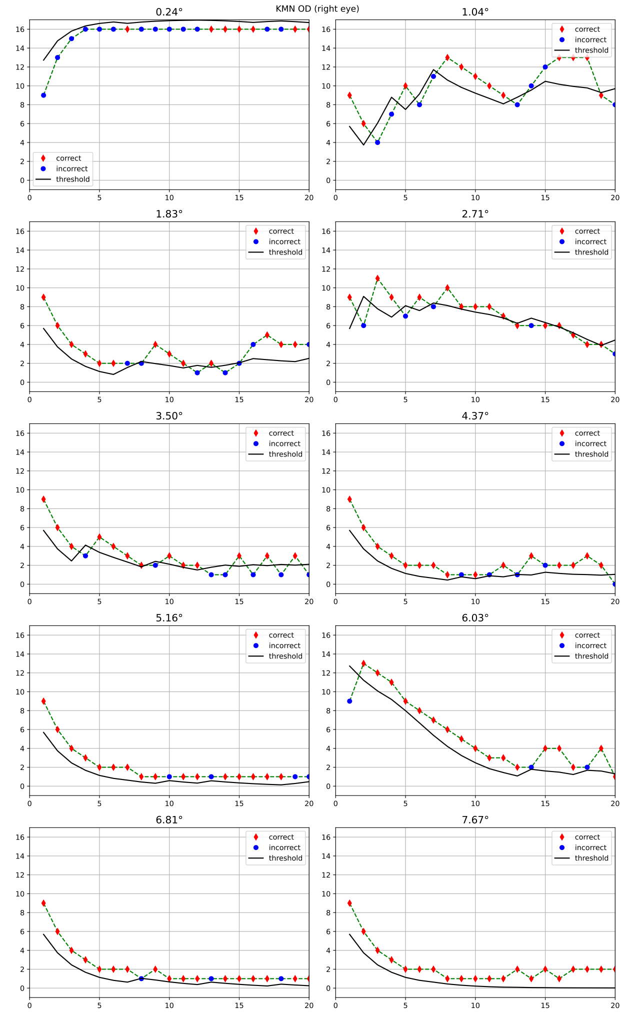

(a) OD (right eye)

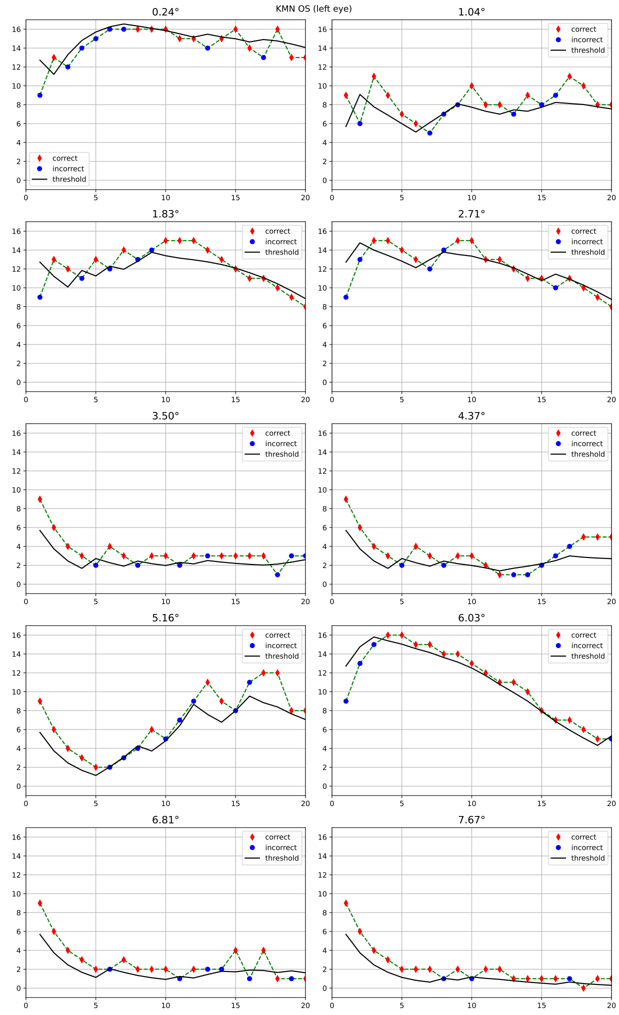

(b) OS (left eye)

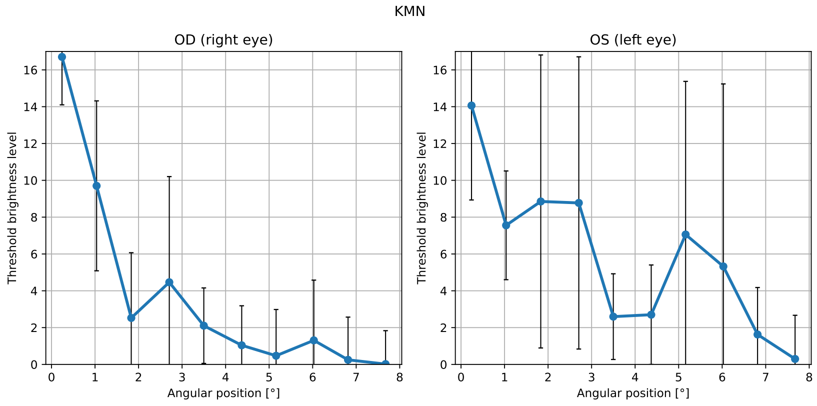

(c) radial plot

6 KPN

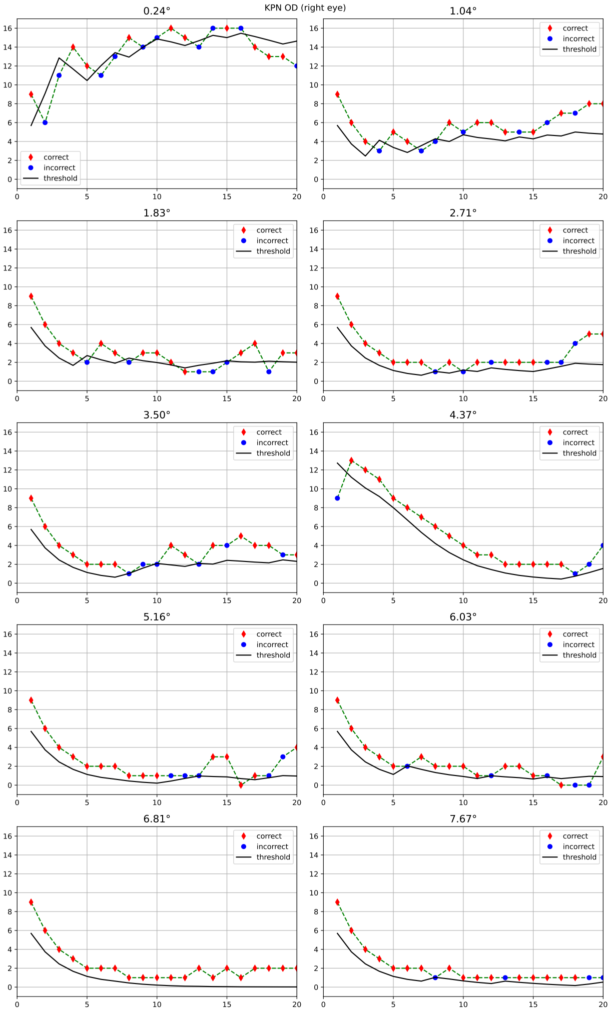

(a) OD (right eye)

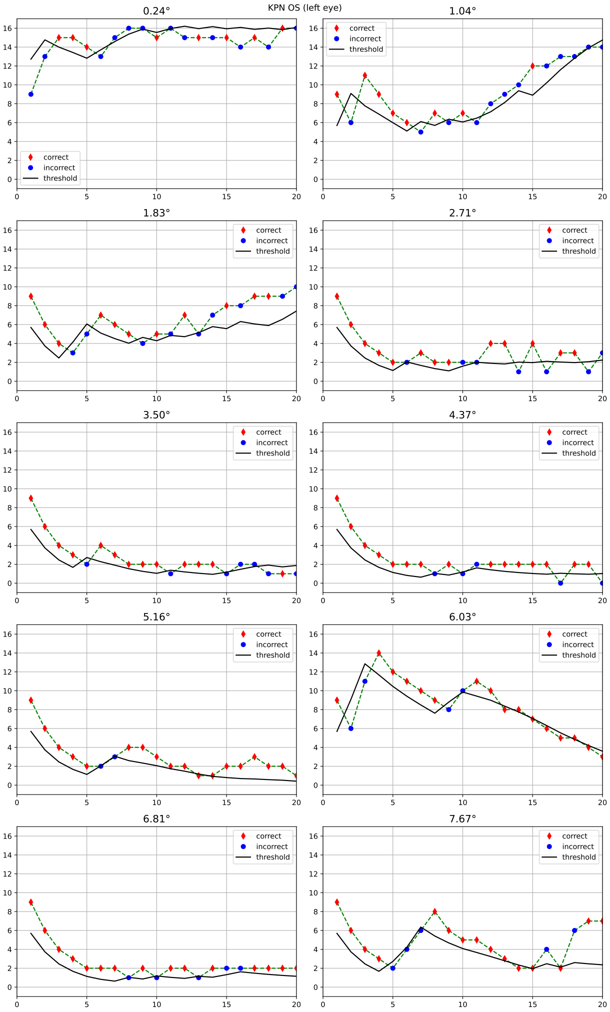

(b) OS (left eye)

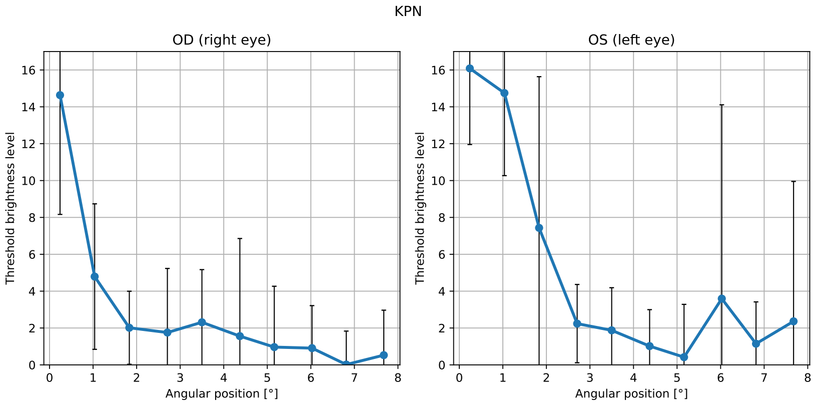

(c) radial plot

7 LYS

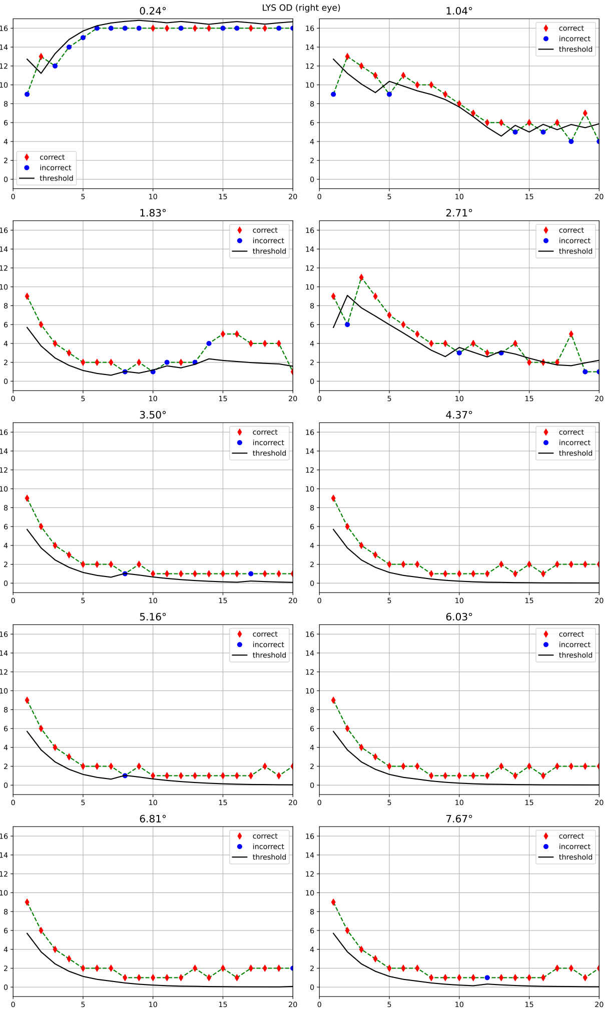

(a) OD (right eye)

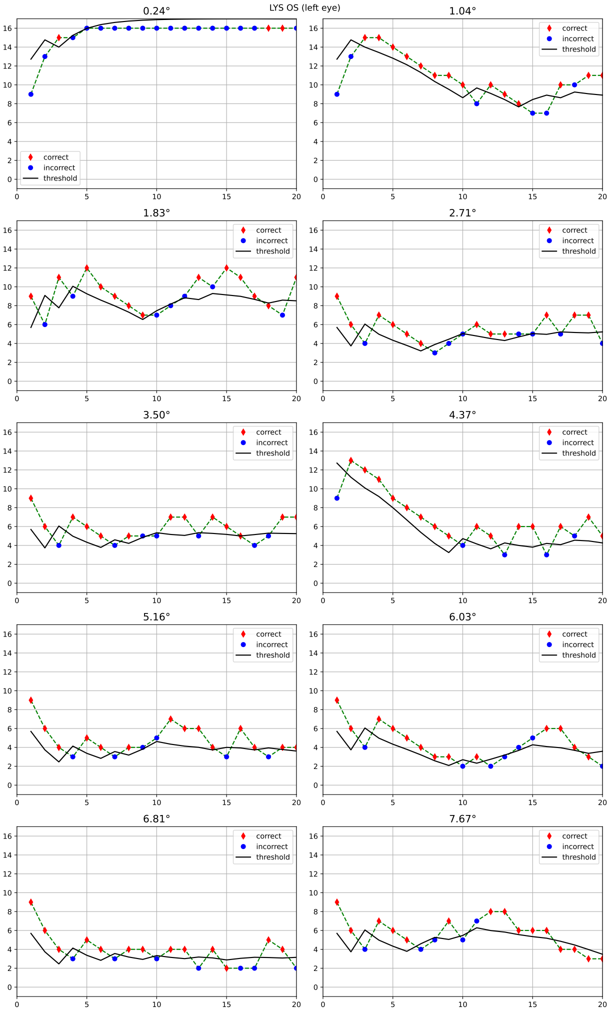

(b) OS (left eye)

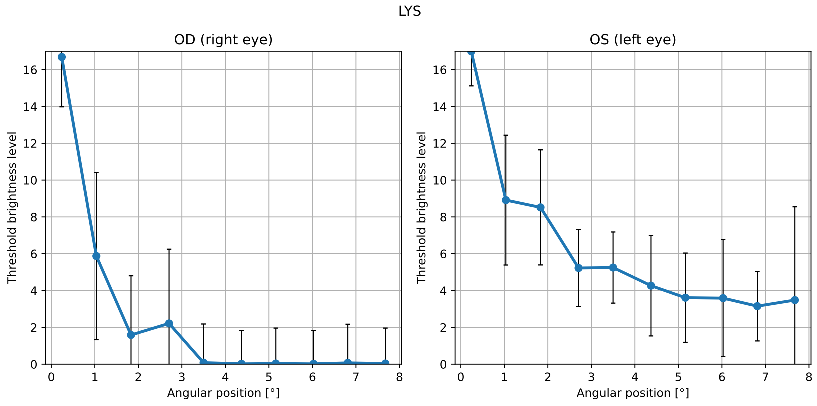

(c) radial plot

8 PDW

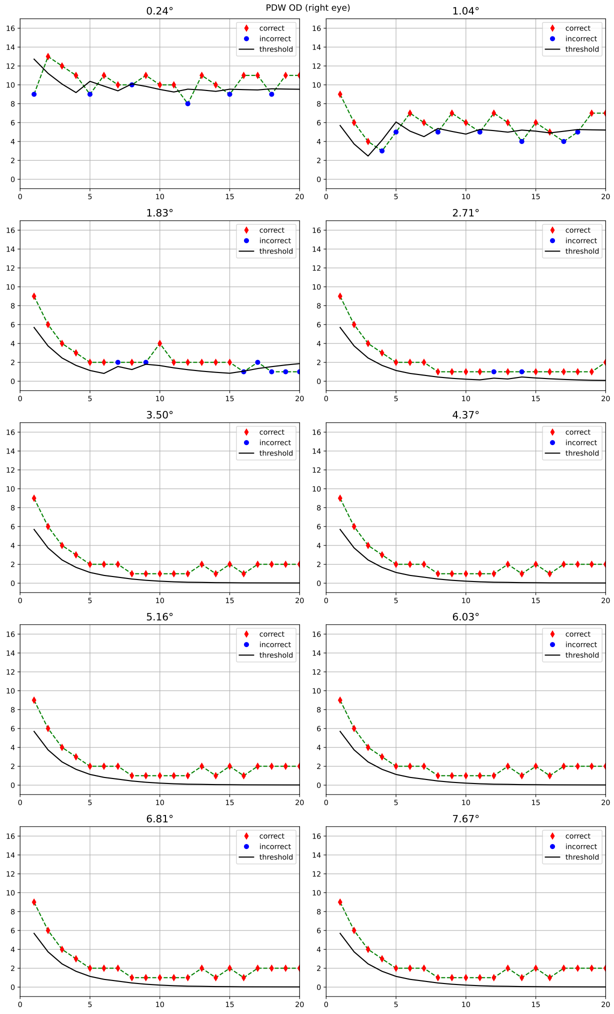

(a) OD (right eye)

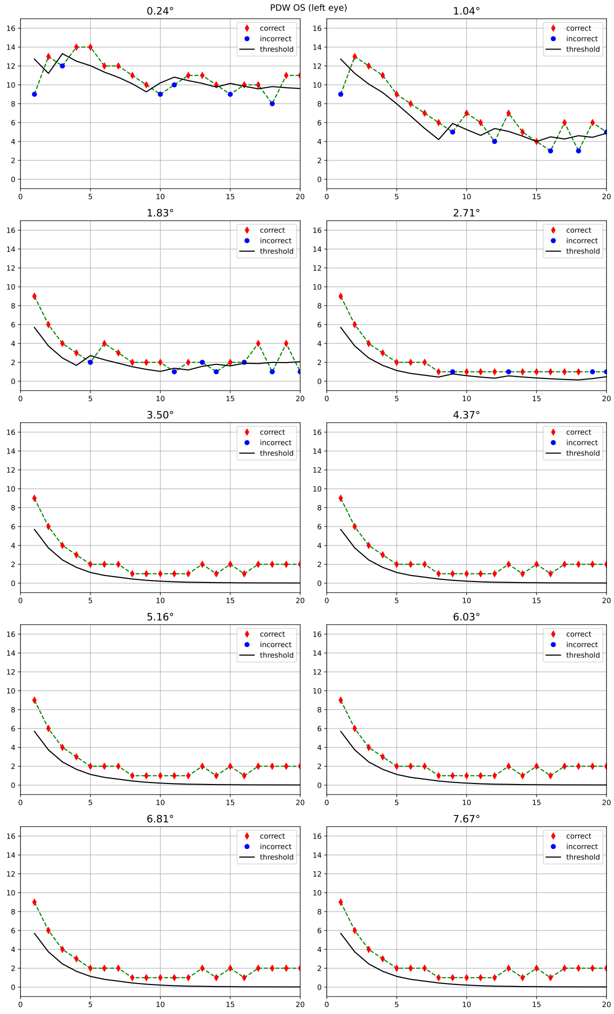

(b) OS (left eye)

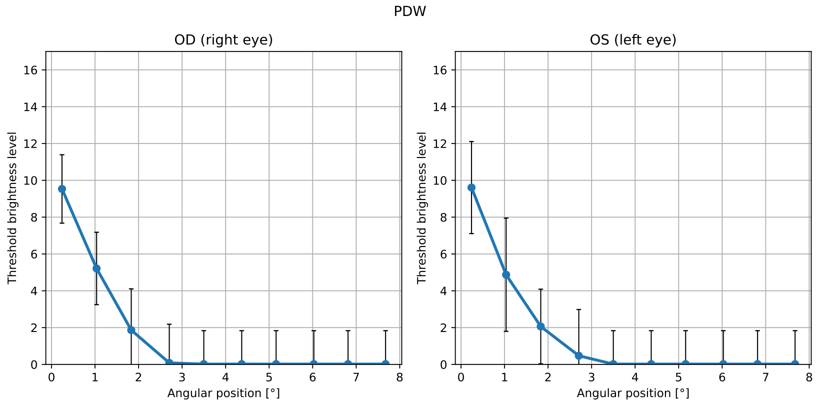

(c) radial plot

9 PTL

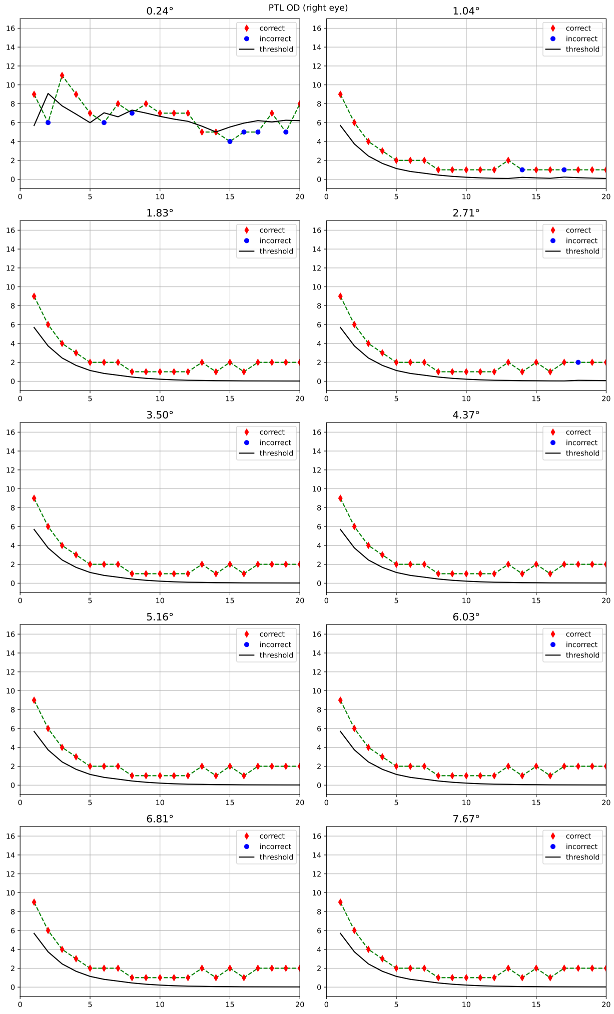

(a) OD (right eye)

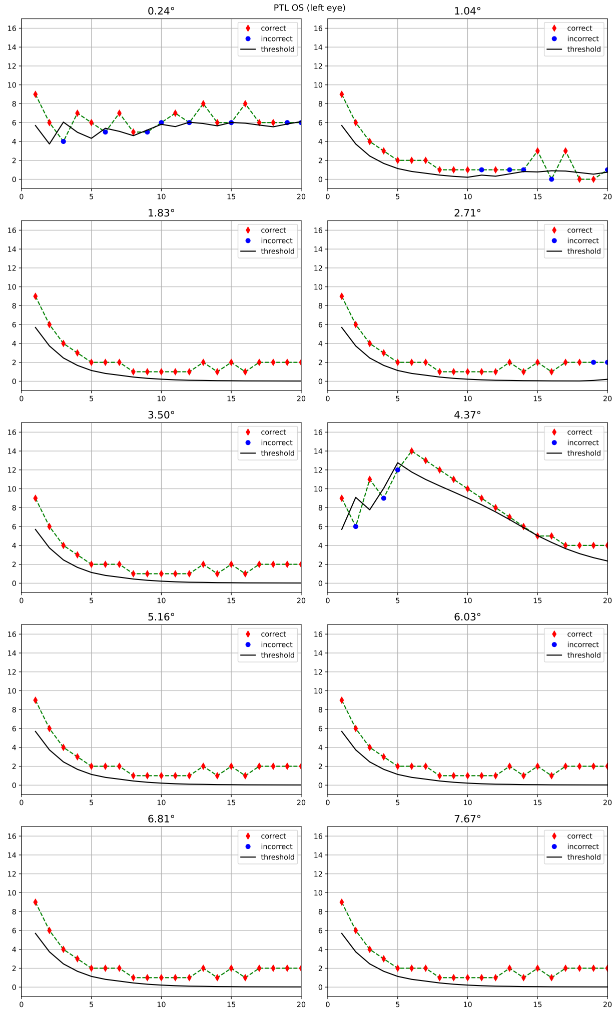

(b) OS (left eye)

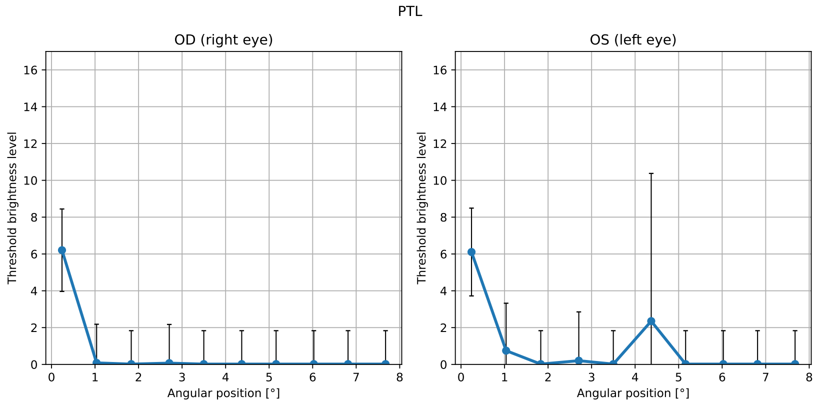

(c) radial plot

10 RGZ

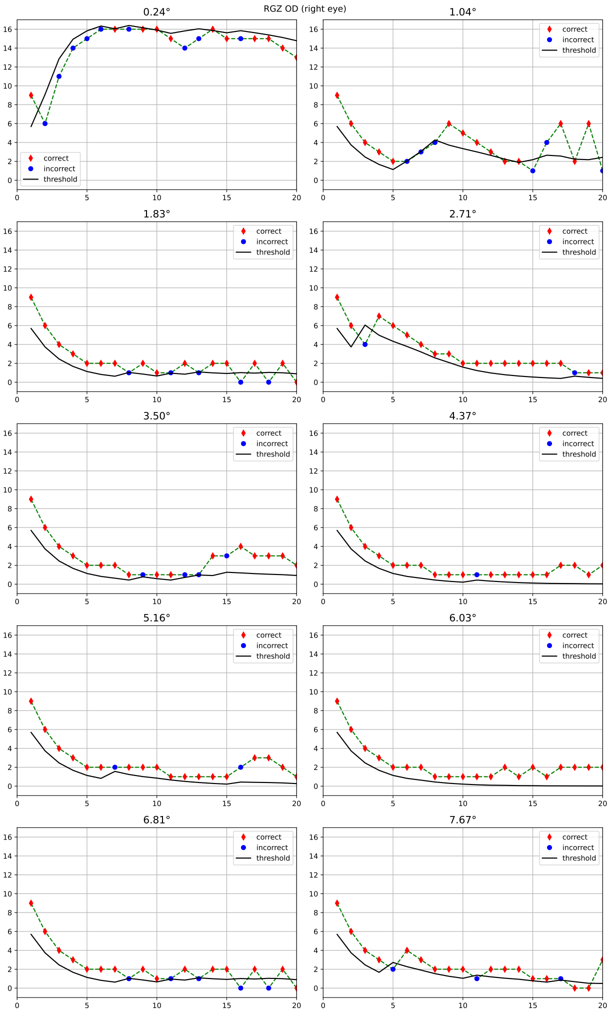

(a) OD (right eye)

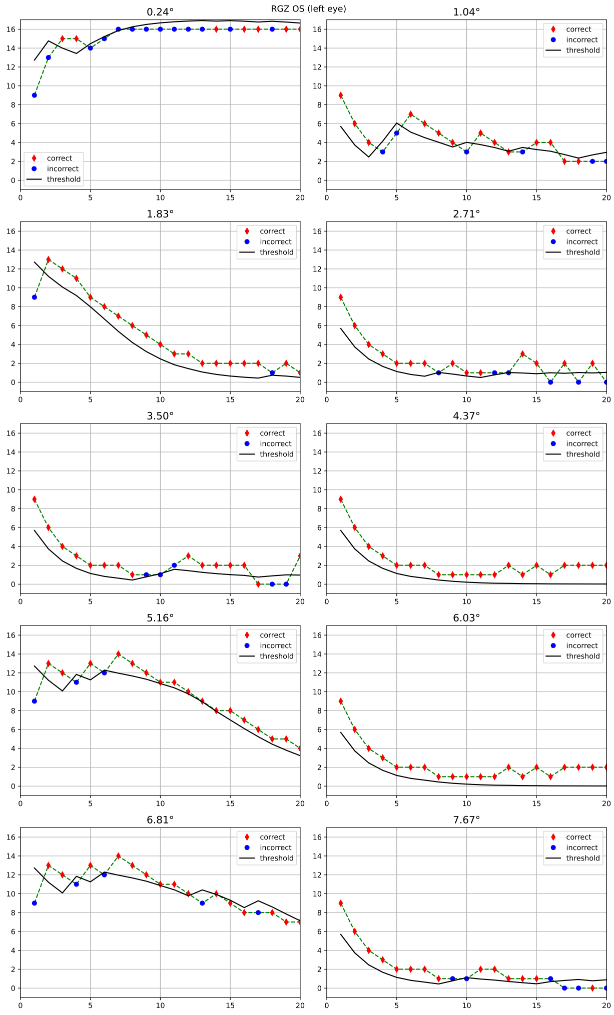

(b) OS (left eye)

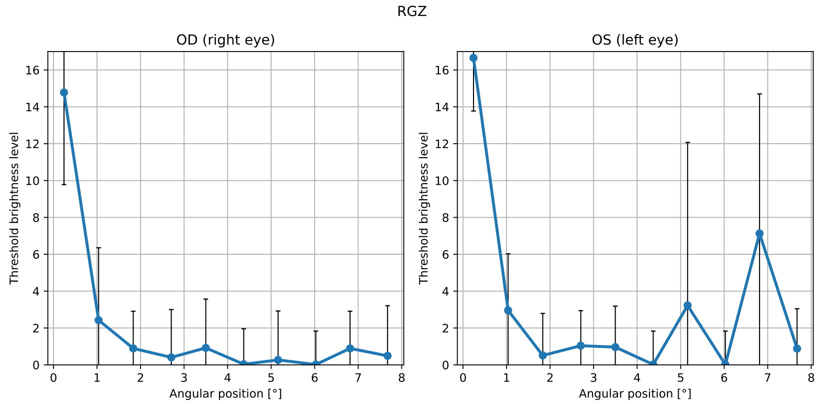

(c) radial plot

11 TCZ

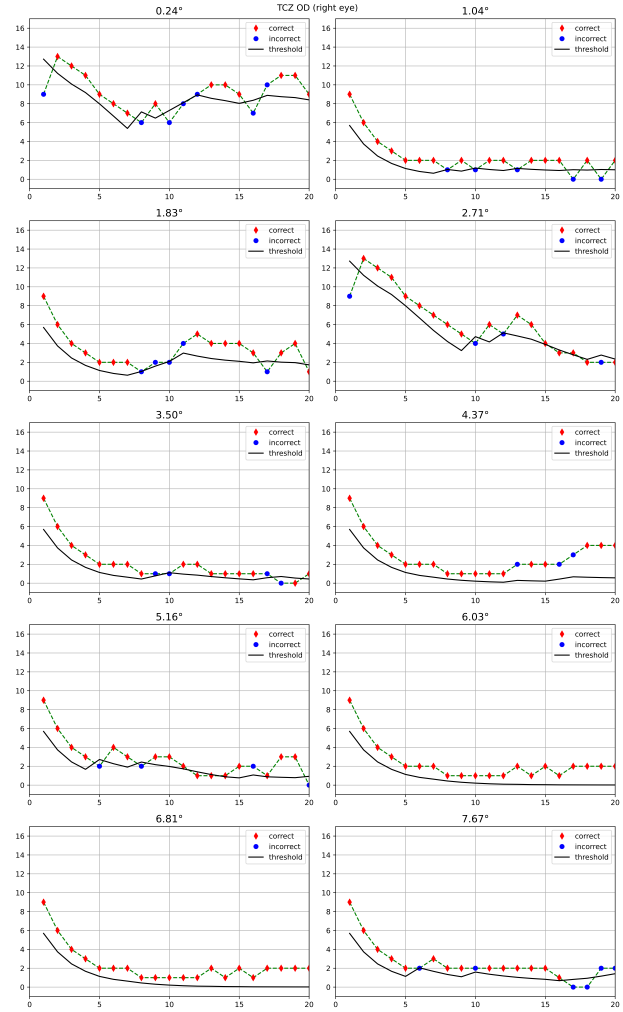

(a) OD (right eye)

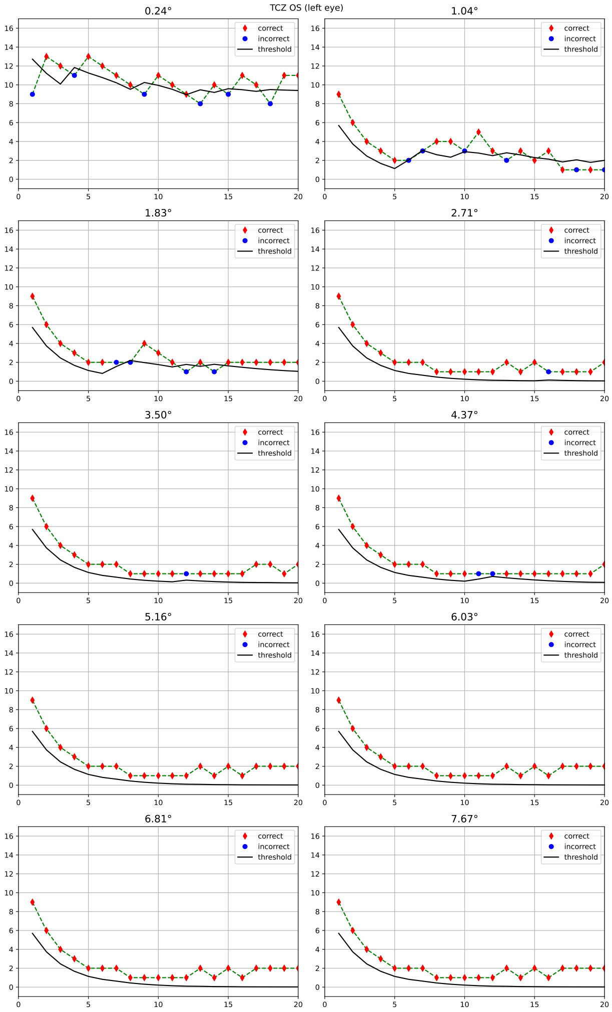

(b) OS (left eye)

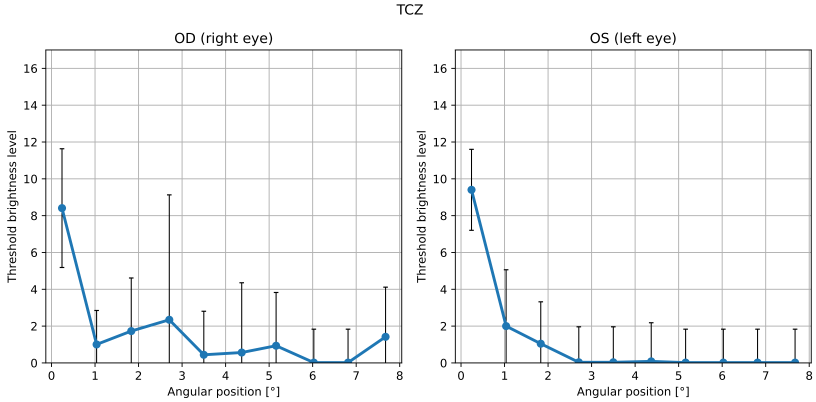

(c) radial plot

12 WJD

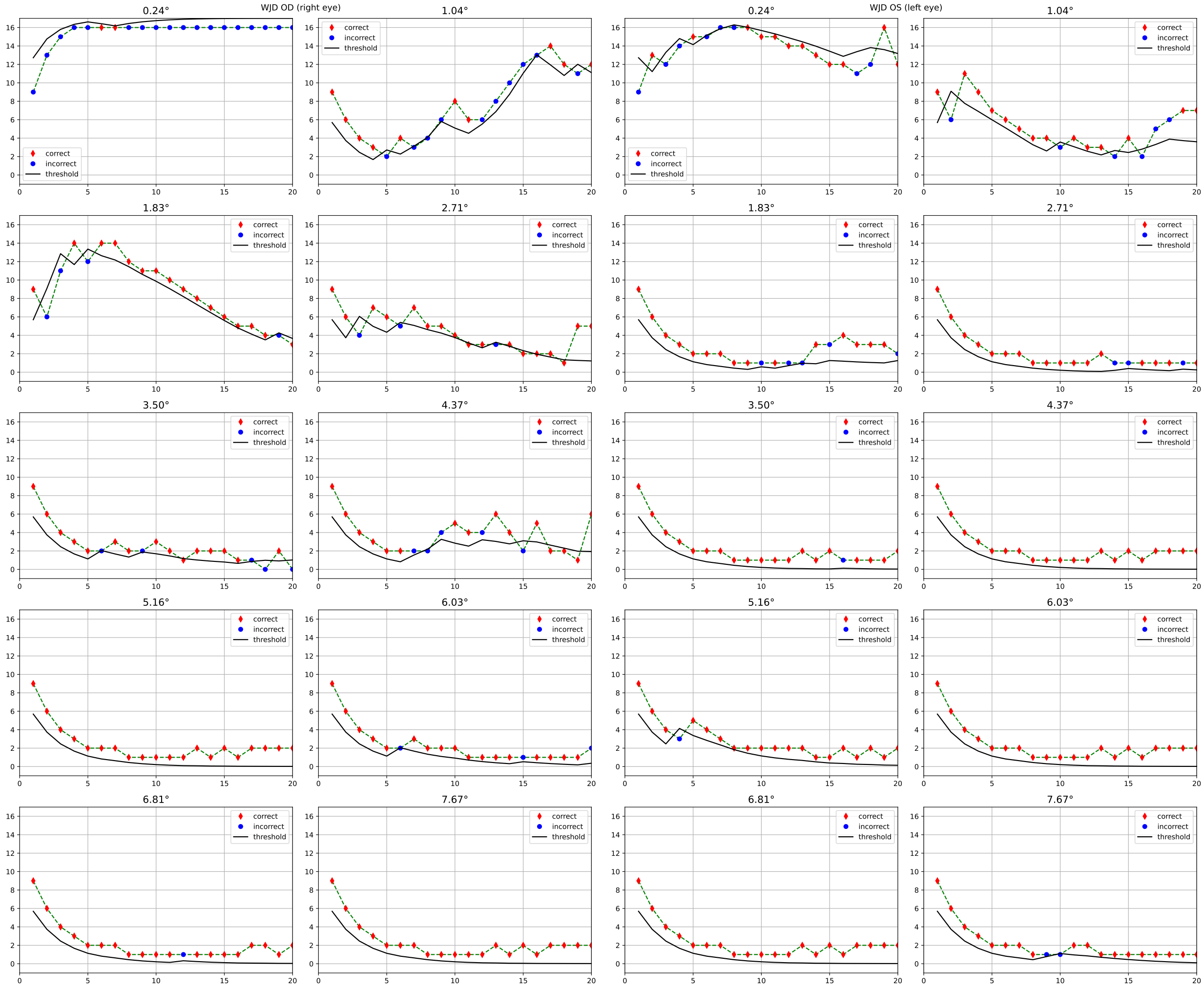

(a) OD (right eye) (b) OS (left eye)

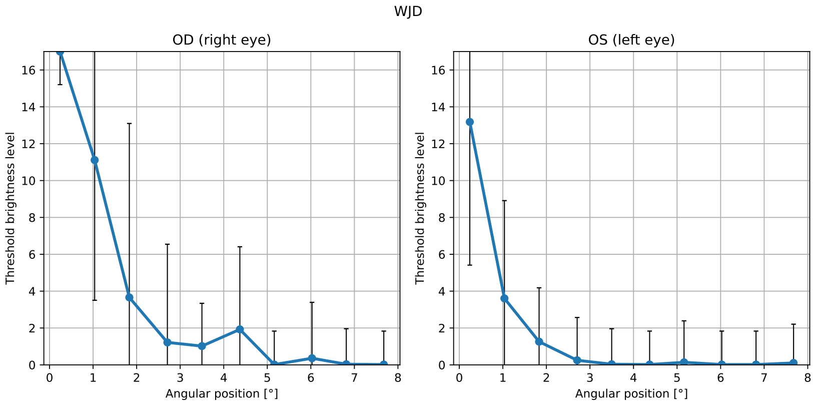

(c) radial plot
