# Supplementary material for: Use of the perceptual point-spread function to assess dysphotopsias
Source: PLoS One. 2024 Jul 19;19(7):e0306331. doi: 10.1371/journal.pone.0306331 (PMC11259305; doi:10.1371/journal.pone.0306331)
Supplement: S2 File — (ZIP) [file pone.0306331.s002.zip › S2_Legend.pdf]

S2_File.zip

Supporting Information to PLOS ONE publication
Use of the perceptual point-spread function to assess dysphotopsias

File 'S2_File.zip' contains CSV files with all data presented in the manuscript. Figure numbers correspond to figures in the manuscript. The contents of the file are as follows:

- This legend:
  - S2_Legend.pdf
- Initials and ages of participants of brightness levels calibration procedure:
  - brightness_calibration_participants.csv
- Data for graphs depicting spectral irradiance of central and probe LEDs irradiances (Fig. 2):
  - led_spectral_irradiance.csv
- Data for graphs depicting brightness level calibration (Fig. 5):
  - cdf_vs_ref_stimulus.csv
  - counts_vs_ref_stimulus.csv
  - new_vs_ref_stimulus.csv
- Data for graphs of courses of individual trials for every angular position (Fig. 6 and graphs from S1_File.pdf):
  - CODE_EYE_NUM_ANGLEdeg.csv

  where CODE – three letter patient designator, EYE – OD or OS, NUM – number of trial, ANGLE – angular position of diode in the given trial.
- Data for radial graphs of pPSFs (Fig. 7 and graphs from S1_File.pdf):
  - CODE_radial_EYE.csv

The CSV files contain header with descriptions of data in columns corresponding to the quantities presented in the manuscript.
